# Longitudinal Metabolomic Profiling of Amino Acids and Lipids across Healthy Pregnancy

**DOI:** 10.1371/journal.pone.0145794

**Published:** 2015-12-30

**Authors:** Karen L. Lindsay, Christian Hellmuth, Olaf Uhl, Claudia Buss, Pathik D. Wadhwa, Berthold Koletzko, Sonja Entringer

**Affiliations:** 1 Development, Health and Disease Research Program, University of California Irvine, School of Medicine, Irvine, California, 92697, United States of America; 2 Ludwig-Maximillian-University München, Division of Metabolic and Nutritional Medicine, Dr. von Hauner Children’s Hospital, University of Munich Medical Centre, Lindwurmstrasse 4, D-80337, Munich, Germany; 3 Institute of Medical Psychology, Charité University Medicine Berlin, Berlin, Germany; The University of Manchester, UNITED KINGDOM

## Abstract

Pregnancy is characterized by a complexity of metabolic processes that may impact fetal development and ultimately, infant health outcomes. However, our understanding of whole body maternal and fetal metabolism during this critical life stage remains incomplete. The objective of this study is to utilize metabolomics to profile longitudinal patterns of fasting maternal metabolites among a cohort of non-diabetic, healthy pregnant women in order to advance our understanding of changes in protein and lipid concentrations across gestation, the biochemical pathways by which they are metabolized and to describe variation in maternal metabolites between ethnic groups. Among 160 pregnant women, amino acids, tricarboxylic acid (TCA) cycle intermediates, keto-bodies and non-esterified fatty acids were detected by liquid chromatography coupled with mass spectrometry, while polar lipids were detected through flow-injected mass spectrometry. The maternal plasma concentration of several essential and non-essential amino acids, long-chain polyunsaturated fatty acids, free carnitine, acetylcarnitine, phosphatidylcholines and sphingomyelins significantly decreased across pregnancy. Concentrations of several TCA intermediates increase as pregnancy progresses, as well as the keto-body β-hydroxybutyrate. Ratios of specific acylcarnitines used as indicators of metabolic pathways suggest a decreased beta-oxidation rate and increased carnitine palmitoyltransferase-1 enzyme activity with advancing gestation. Decreasing amino acid concentrations likely reflects placental uptake and tissue biosynthesis. The absence of any increase in plasma non-esterified fatty acids is unexpected in the catabolic phase of later pregnancy and may reflect enhanced placental fatty acid uptake and utilization for fetal tissue growth. While it appears that energy production through the TCA cycle increases as pregnancy progresses, decreasing patterns of free carnitine and acetylcarnitine as well as increased carnitine palmitoyltransferase-1 rate and β-hydroxybutyrate levels suggest a concomitant upregulation of ketogenesis to ensure sufficient energy supply in the fasting state. Several differences in metabolomic profiles between Hispanic and non-Hispanic women demonstrate phenotypic variations in prenatal metabolism which should be considered in future studies.

## Introduction

The state of pregnancy is associated with many complex metabolic alterations in order to meet not only the energetic demands of the developing fetus but also ensure deposition of adequate energy substrate stores to contribute to neonatal nutritional requirements following birth. Glucose, amino acids (AA) and lipids are all utilized by the fetoplacental unit, and thus maternal metabolism must adapt, in both the fasting and fed state, to ensure the fetal demands are met while also ensuring an adequate maternal nutrient supply to cope with the increased physiological demands of pregnancy and of labor and lactation. For instance, enhanced ketogenesis during fasting serves to meet energy demands while minimizing protein catabolism. Maternal plasma AA levels are noted to fall during pregnancy [1], related to increased placental uptake, increased insulin levels, hepatic diversion for gluconeogenesis and uptake by the fetus for use in protein synthesis and oxidation [2].

To date, our knowledge of alterations in these metabolic processes during pregnancy is generated from experimental studies in small populations that have relied on specific nutritional biomarkers to gain an understanding of individual nutrient metabolism [1–5]. Although there is general consensus that the normal progressive insulin resistance of pregnancy is an underlying driver for many of the observed metabolic adaptations, our understanding remains incomplete regarding the specific nutrients, mechanisms and hormonal processes involved.

Metabolomics technology offers enormous potential to increase our knowledge of subtle changes in maternal metabolism in pregnancy and their relation to maternal nutritional status and diet in a comprehensive manner. Two recent cross-sectional studies have reported on the plasma metabolomic profiles of healthy pregnant women [6,7] while a further study examined urinary metabolomics [8], each using an untargeted analytical approach. While this method offers potential for a broad range of metabolites to be explored, meaningful interpretation of the data may be limited by non-specificity for any given metabolite class, and thus it only serves non-hypothesis driven studies [9]. In contrast, targeted metabolomic techniques facilitate the profiling of specific metabolites of interest in a given population, to aid in-depth analysis of metabolic processes in the context of a pre-formed hypothesis. A further limitation of the recent metabolomic studies in healthy pregnant cohorts was that each employed a cross-sectional design and therefore had different subjects representing each stage of pregnancy. This precludes reliable assessment of longitudinal changes in metabolism throughout gestation, as individual genetic or phenotypic characteristics between the subgroups across gestational windows may have influenced metabolite concentrations. For example, significant differences in metabolomics profiles have been reported between non-pregnant populations from different geographical locations, which may be attributed to genetic, dietary or gut microbial variations [10].

Thus, there remains a need to utilize the recent advancements in metabolomics technology to characterize maternal metabolism across normal, healthy pregnancy while also considering the influence of inter-individual variations. Increased knowledge in this field would aid our understanding of the many complex metabolic changes that occur across gestation, paving the way for future studies to relate such changes and trajectories to various maternal, fetal and infant factors. The present study aims to fill this gap through the use of targeted metabolomic technology and a longitudinal design throughout gestation in a relatively large cohort of healthy, non-diabetic women. The targeted approach facilitates a specific focus on nutritional metabolites of interest, namely proteins and lipids, as we hypothesize that distinct changes in concentrations will occur as gestation advances to reflect the shift from the early pregnancy anabolic phase to later pregnancy fatty acid catabolism. The present analysis also quantifies intermediary metabolites generated through carbohydrate, protein and fat metabolism in order to deepen our understanding of which nutrients serve as important energy substrates across gestation. Furthermore, the potential influence of maternal ethnicity on metabolomic profiles was considered in this sample which predominantly consisted of Hispanic and Non-Hispanic White pregnant women.

## Materials and Methods

This was a secondary analysis conducted within a larger longitudinal, prospective cohort study designed to investigate the interplay between maternal prenatal stress and offspring development at the University of California, Irvine (UCI). The parent study enrolled pregnant women attending antenatal care at clinics affiliated with the UCI Medical Center, in Orange County, California between 2011 and 2015, and the present analysis included 167 women enrolled between March 2011 and December 2013. Women were eligible for inclusion if they were >18 yrs age with a singleton, intrauterine pregnancy and non-diabetic. The University of California’s Institutional Review Board approved the protocol and written, informed consent was obtained from each participant.

All participants were recruited in the first trimester and prospectively followed until the end of their pregnancy. Women were invited to attend three prenatal visits for the study, once in each trimester, which occurred at a mean ± standard deviation gestational age of 13.1±1.8 weeks, 20.5±1.4 weeks and 30.5±1.4 weeks, respectively. Race/ethnicity of the participants was assessed by self-report at the first study sample. The racial-ethnic breakdown of the study sample is described in Table 1. Each visit occurred in the morning, following an overnight fast, for blood sample collection. A 10ml EDTA tube was drawn by standard venipuncture technique by a trained phlebotomist and then centrifuged for 15 min at 1200 g. Separated plasma aliquots of 0.5ml were transferred to 2ml screw top plastic vials, labeled and stored at -80°C until analysis. The measurement of polar lipids, AA, tricarboxylic acid (TCA) cycle intermediates, keto-bodies and NEFA using targeted metabolomic profiling techniques is described below.

**Table 1:** Subject demographics (N = 160).

	**Mean (SD)**
Maternal age (years)	27.6 (5.5)
Pre-pregnancy weight (kg)	69.0 (16.9)
Height (cm)	163.2 (6.8)
Pre-pregnancy BMI (kg/m^2^)	25.9 (6.1)
	**N (%)**
Race/Ethnicity:	
Hispanic White	51 (31.7)
Hispanic Asian	2 (1.2)
Hispanic Other	15 (9.4)
Non-Hispanic White	68 (42.2)
Non-Hispanic Black	4 (2.5)
Non-Hispanic Asian	12 (7.5)
Non-Hispanic Multi-race	7 (4.3)
Primiparous	67 (41.9)
Prenatal multivitamin supplement use at any stage in pregnancy	141 (88.1)

BMI, body mass index; SD, standard deviation

### Amino acids

Aliquots of 10 μL plasma were prepared using derivatization as previously reported [11]. For internal standardization, a labeled amino acid standards set (set A, Cambridge Isotope Laboratories) was mixed with L-Asparagine (15N2, 98%, Cambridge Isotope Laboratories) and L-Tryptophan (Indole-D5, 98%, Cambridge Isotope Laboratories) and added to the precipitation reagent. AA butylester were determined by ion-pair liquid chromatography coupled to mass spectrometry detection (LC-MS/MS). 10 μL of the prepared sample were injected into the HPLC system (HPLC 1100, Agilent, Waldbronn, Germany) and chromatographic separation was performed with a XBridge C18 column (Waters GmbH, Eschborn, Germany). MS detection was performed with an API 2000 triple quadrupole instrument (Sciex, Darmstadt, Germany) with an APCI source operating in positive ion ionization mode. Data acquisition on the mass spectrometer was controlled by Analyst 1.6.2 software (AB Sciex, Darmstadt, Germany). Data handling and quantification were also performed with Analyst 1.6.2 software (AB Sciex, Darmstadt, Germany). The sums of non-essential and essential AA were computed in addition to the sum of the branched chain AA (BCAA) leucine, isoleucine and valine.

### TCA Intermediates

Metabolites of the TCA cycle and keto-acids were measured by a modified LC-MS/MS method based on previously published methods [12,13]. In detail, proteins of 20 μL plasma were precipitated by adding 200 μL acetonitrile with 0.1% formic acid including D_3_-methylmalonic acid as internal standard. After centrifugation and cooling (20min at -20°C), 100 μL of the supernatant were evaporated to dryness and re-suspended in 50 μL water. Fife μL of the extracted samples were injected to an Agilent 1200 HPLC and molecular species were separated on a Kinetex F5 core-shell HPLC column, 150 x 2.1 mm, 2.6 μm particle size (Phenomenex, Aschaffenburg, Germany). The mobile phase A was water with 1% formic acid and mobile phase B was composed of methanol/ isopropanol (50:50) with 1% formic acid. The gradient elution at a flow rate of 200 μL/ min was from 1% B to 85% B within 9 minutes and turned back to initial conditions of 1%B within 1 minute. Re-equilibration was held for 5 minutes at 1% B. The triple quadrupole mass spectrometer (AB Sciex API4000; Applied Biosystems, Darmstadt, Germany) was operated in negative scheduled multiple reaction monitoring mode using electrospray ionization (ESI).

### Non-esterified fatty acids

Analysis for NEFA was performed as previously reported [14]. Briefly, 20 μl plasma were mixed with 200 μl isopropanol containing uniformly labelled palmitic acid (U-13C16, 98%, Euriso-Top). After centrifugation the supernatant was transferred for LC-MS/ MS analysis. An UPLC diphenyl column (Pursuit UPS Diphenyl, Varian, Darmstadt, Germany) was used for chromatographic separation with an Agilent 1200 SL series HPLC system (Waldbronn, Germany). The injection volume was set to 10 μL with an eluent flow rate of 700 mL/min. A hybrid triple quadrupole mass spectrometer (4000 QTRAP, AB Sciex, Darmstadt, Germany) operating in negative ESI mode was coupled to the HPLC system for identification of NEFA. With the analytical method applied, fatty acids (FA) are separated according to chain length and number of double bonds, but not according to position of double bonds. NEFA are mentioned as CX:Y. In this nomenclature, X is the length of the carbon chain, Y is the number of double bonds. NEFA sub-groups according to level of saturation were created and the constituents summed.

### Polar Lipids

Flow-injection mass spectrometry (FIA-MS/MS) was used to analyze polar lipids. Ten μl plasma or 10 μl standard solution were diluted with 500 μl methanol, containing internal standards for different lipid groups and ammonium-acetate. D3-Carnitine C2, D3-Carnitine C8, D3-Carnitine C16, 13C6-D-Glucose, (all Cambridge Isotope Laboratories), Lyso-PC(13:0), and PC(14:0/14:0) (both Avanti Polar Lipids) were used as internal standards. After centrifugation, 200 μL of the centrifuged supernatant was mixed with 700 μl methanol and then used for FIA-MS/MS analysis. Samples were analyzed with a triple quadrupole mass spectrometer (QTRAP4000, Sciex, Darmstadt, Germany) with an ESI source, which was used in both positive and negative mode. The MS was coupled to a LC system (Agilent, Waldbronn, Germany). MS/MS analysis was run in Multiple Reaction Monitoring (MRM) mode. Analyst 1.6.2 software, followed by in-house processing with the statistical program R (R Project for Statistical Computing, http://www.r-project.org/) was used to post-process the entire analytical process.

The analysis comprised acylcarnitines (Carn), diacyl-phosphatidylcholines (PCaa), acyl-alkyl-phosphatidylcholines (plasmalogens, PCae), sphingomyelins (SM), lysophospholipids (lyso-PL) and sum of hexoses which is further mentioned as glucose presenting more than 80% of this value. As a point to note, the analytical technique applied here is not capable of determining the position of the double bonds and the distribution of carbon atoms between fatty acid side chains. The polar lipids are mentioned as CX:Y. In this nomenclature, X is the length of the carbon chain, Y is the number of double bonds, “a” indicates that the acyl chain is bound via an ester bond to the backbone, while “e” means binding by an ether bond.

Carn sub-groups were created and summed according to chain lengths while each of the remaining polar lipid families were grouped and their total concentrations summed.

The following ratios of metabolites were also computed as indicators of metabolic pathways: ratio of various acylcarnitines (C14.0, C16.0, C18.0, C18.1, C18.2) to free carnitine as an indicator of carnitine palmitoyltransferase (CPT)-1 rate; ratio of Carn.a.C2 to each of the acylcarnitines C14.0, C16.0, C18.0, C18.1, C18.2 as an indicator of β-oxidation rate.

All metabolites are reported in units of μmol/L plasma.

### Quality Control

Six aliquots of a plasma quality control (QC) sample were consistently measured between samples of one batch to ensure correct quantification of the analytes. The QC criterion was defined as inter- and intra-batch coefficient of variance of 20%.

As the aim of this longitudinal study was to profile metabolomics among 167 uncomplicated, healthy pregnant women, all data from 7 subjects that were diagnosed with diabetes during their pregnancies (one with type 1, one with type 2 and 5 with gestational diabetes) were excluded. Among the remaining 160 participants, blood samples were collected and analyzed from 142 women at trimester 1, 155 at trimester 2 and 155 at trimester 3. Missing cases within each trimester were due to non-attendance for blood sample collection. Furthermore, data from 15 samples which were collected from participants who presented in the non-fasting state were excluded, resulting in final statistical analysis for N = 135 women at trimester 1, N = 153 at trimester 2 and N = 150 at trimester 3. All participants were followed until delivery to ensure viable pregnancy outcomes. The mean ± standard deviation gestational age at delivery was 39.4±1.4 weeks, and mean birth weight was 3360±522 g. All children were healthy at the time of birth.

Initially, individual outlying values for a given metabolite were detected if >3 times the standard deviation (SD) of the second highest value. Further possible outliers were detected through inspection of boxplots for each metabolite at each time point of measurement and individual outlying values were removed from the dataset as appropriate.

### Statistical Analysis

Statistical analyses were performed using IBM SPSS for Windows, version 22. Normality distributions of metabolites were explored through visual inspection of histograms and many were found to have a non-normal distribution.

Descriptive statistics were used to describe the median and interquartile range (IQR) of individual metabolites, groups of metabolites and metabolic ratios within each trimester for the total population and stratified by Hispanic/non-Hispanic ethnicity. To assist analysis of large volumes of data, the polar lipids lyso-PL, PCaa, PCae and SM were analyzed by sum of their sub-groups rather than as individual metabolites. The non-parametric Mann Whitney U test was used to compare median values between ethnic groups within trimesters and the Wilcoxon Rank test was employed to analyze differences in median values for each parameter between trimesters among the total population and among both ethnic groups. Bonferroni correction was applied to address the problem of multiple comparisons (97 metabolites x 3 timepoints), such that a P-value of 0.05 was divided by 291, to set the level of significance at P<0.00017. Results of total population differences in metabolites and metabolic ratios between trimesters were visualized using histograms.

## Results

Metabolomic analysis quantified the following metabolites: 21 AA which may be classified as essential (leucine, isoleucine, valine, methionine, phenylalanine, tryptophan and threonine) and non-essential (alanine, arginine, asparagine, aspartic acid, glutamine, glutamic acid, glycine, citrulline, ornithine, proline, serine, tyrosine, cysteine and taurine); 21 NEFA which included 7 saturated (C11:0, C12:0, C14:0, C15:0, C16:0, C17:0, C18:0), 5 monounsaturated (C14:1, C16:1, C17:1, C18:1, C20:1) and 9 polyunsaturated (C18:2, C18:3, C20:2, C20:3, C20:4, C20:5, C22:4, C22:5, C22:6) NEFA; 164 polar lipids including sum of hexoses, free carnitine and 15 Carn (3 short-chain (C2-C4), 5 medium-chain (C8-C12) and 7 long-chain (C14-C18) acylcarnitines), 17 lyso-PL, 39 PCaa, 37 PCae, 54 SM; and 14 metabolites involved in fatty acid and amino acid oxidation, among them TCA intermediates, keto acids of BCAA degradation and keto bodies.

Demographics of the study population are presented in Table 1. The mean body mass index (BMI), based on self-reported pre-pregnancy weight, was 26 Kg/m^2^. Forty-two per cent of the population was of Hispanic ethnicity and 42% were primiparous. While 16% reported using nicotine products pre-conception, only 6% reported their use at any stage during pregnancy. The majority of women reported taking any type of prenatal multivitamin supplements, including folic acid, during their pregnancy.

**Fig 1:**
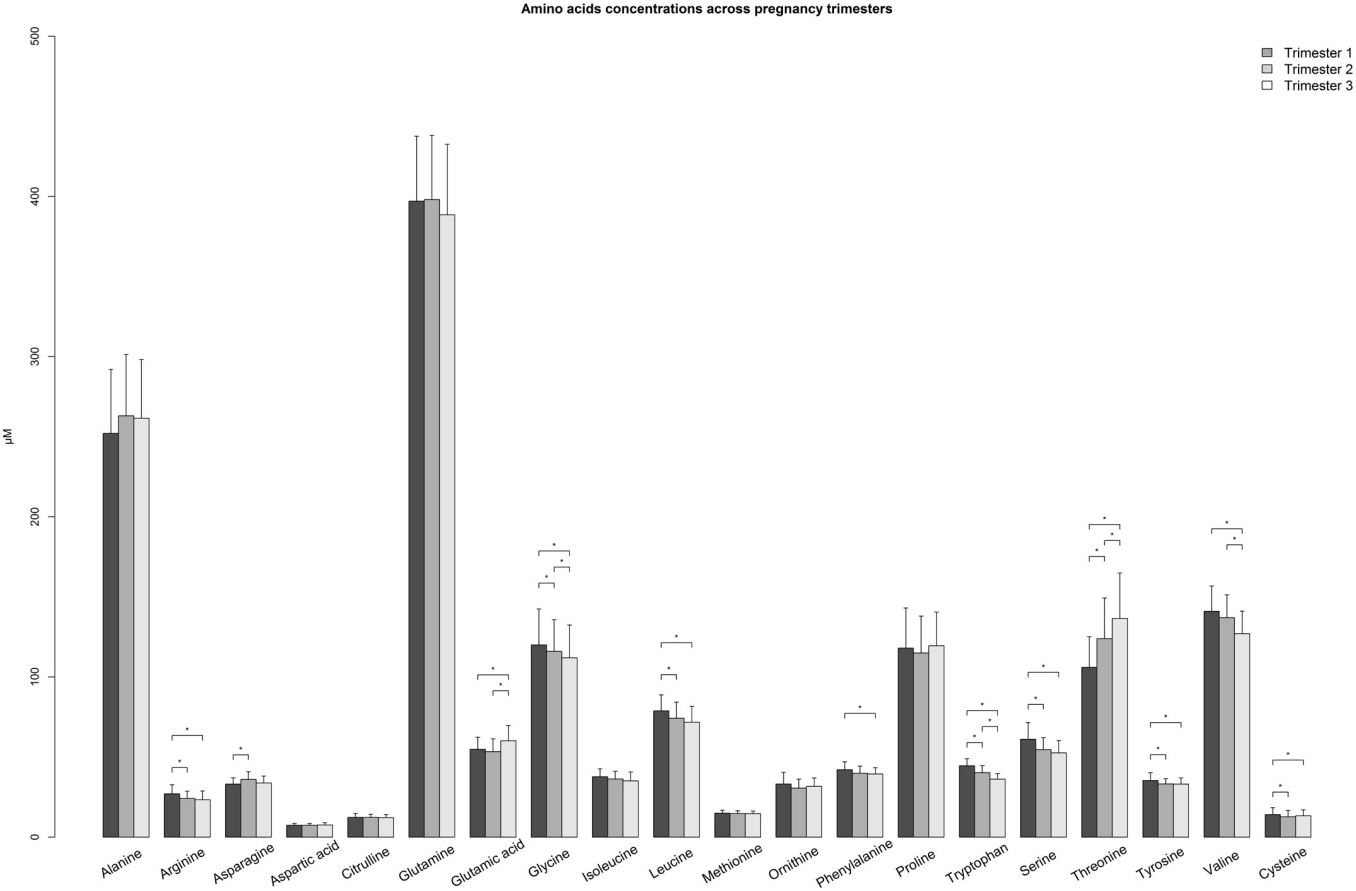
Barplot comparing plasma amino acid concentrations across trimesters among pregnant women. Median (+ Interquartile range/2) was plotted. * p-value < 0.00017, p-value was calculated by Mann-Whitney U Test between trimesters.

**Fig 2:**
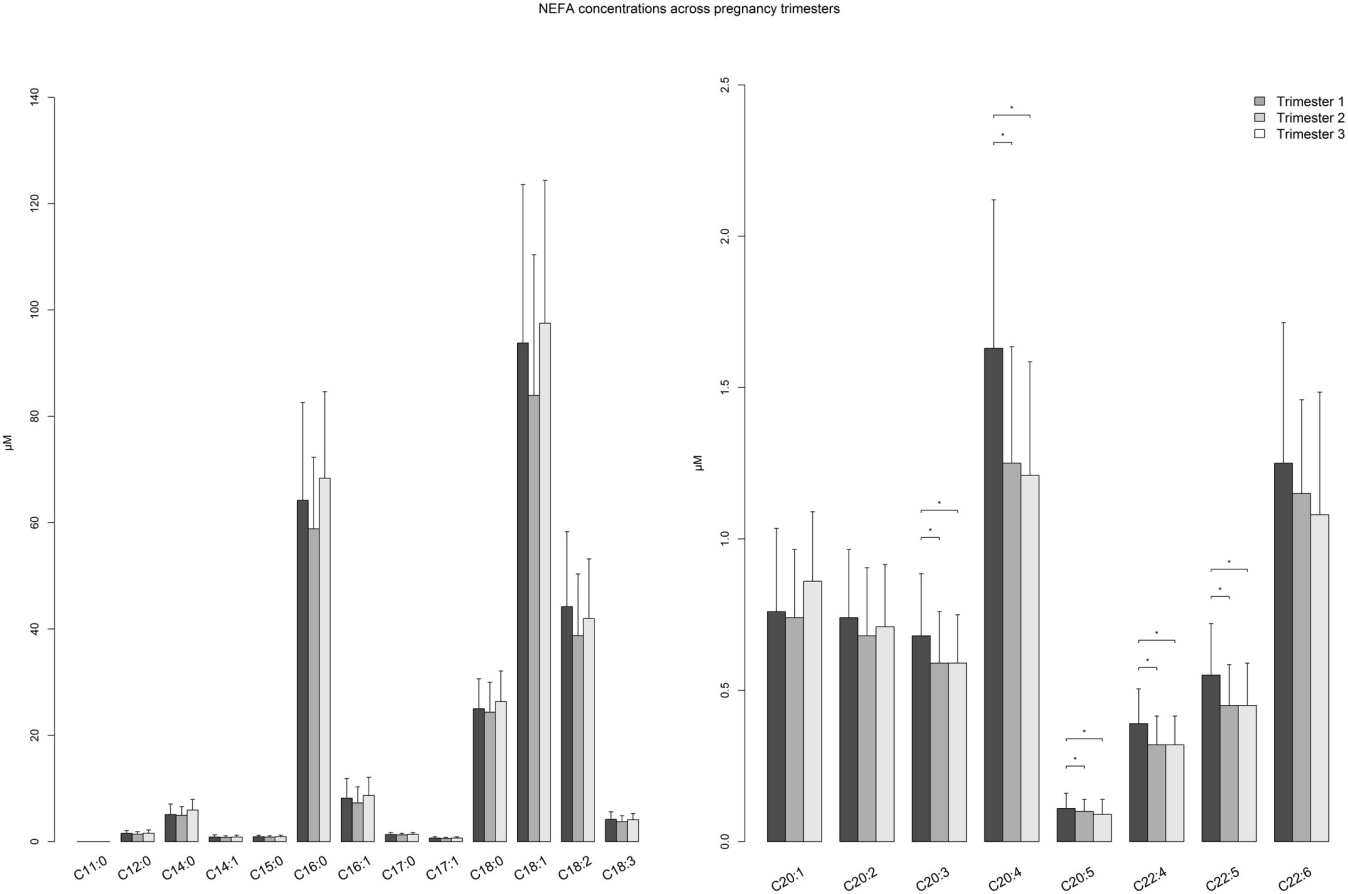
Barplot comparing plasma non-esterified fatty acid concentrations across trimesters among pregnant women. Median (+ Interquartile range/2) was plotted * p-value < 0.00017, p-value was calculated by Mann-Whitney U Test between trimesters.

**Fig 3:**
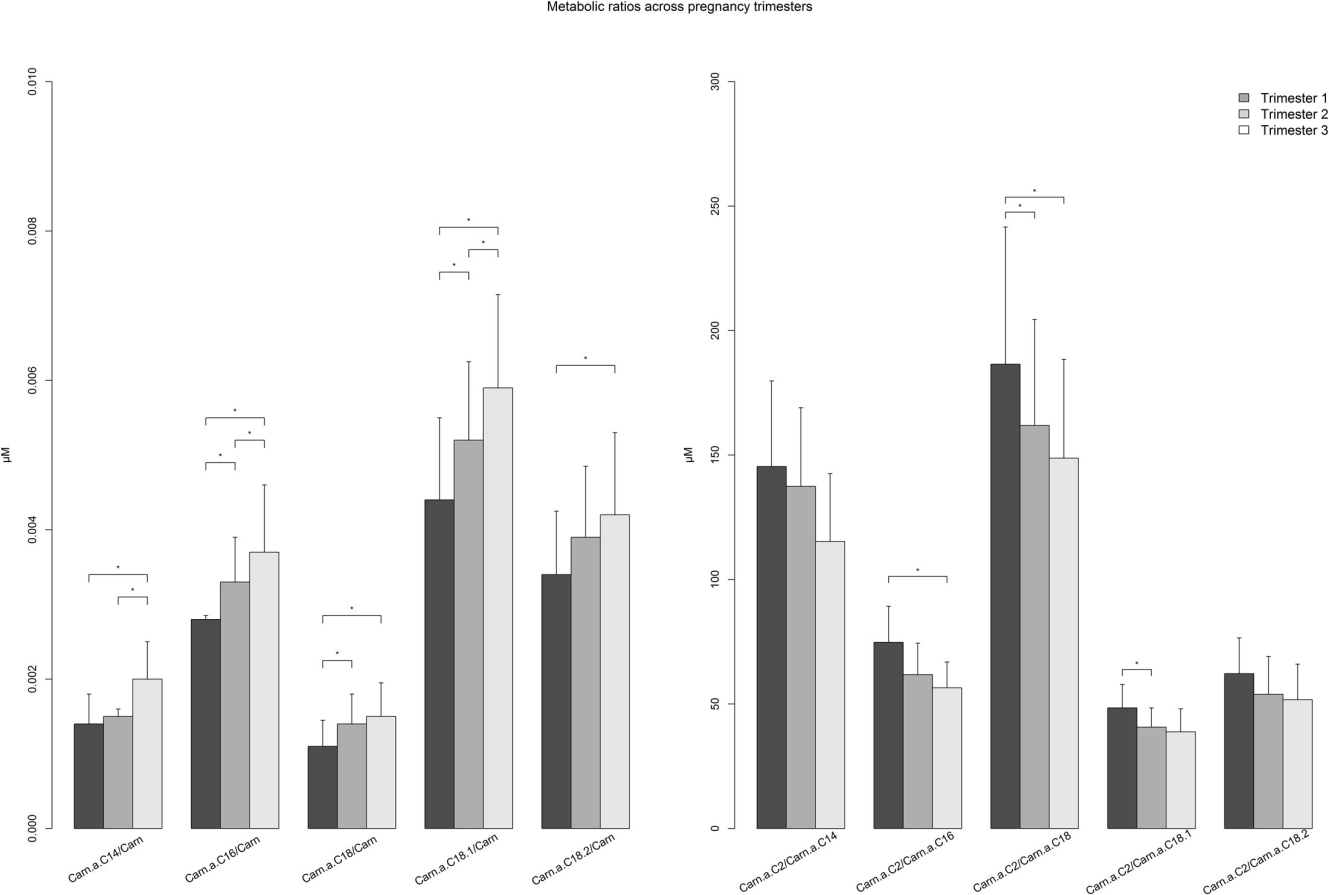
Barplot comparing metabolic ratios as indicators of β-oxidation (right) and carnitine palmitoyl transferase-1 activity (left) across trimesters among pregnant women. Median (+ Interquartile range/2) was plotted * p-value < 0.00017, p-value was calculated by Mann-Whitney U Test between trimesters.

**Fig 4:**
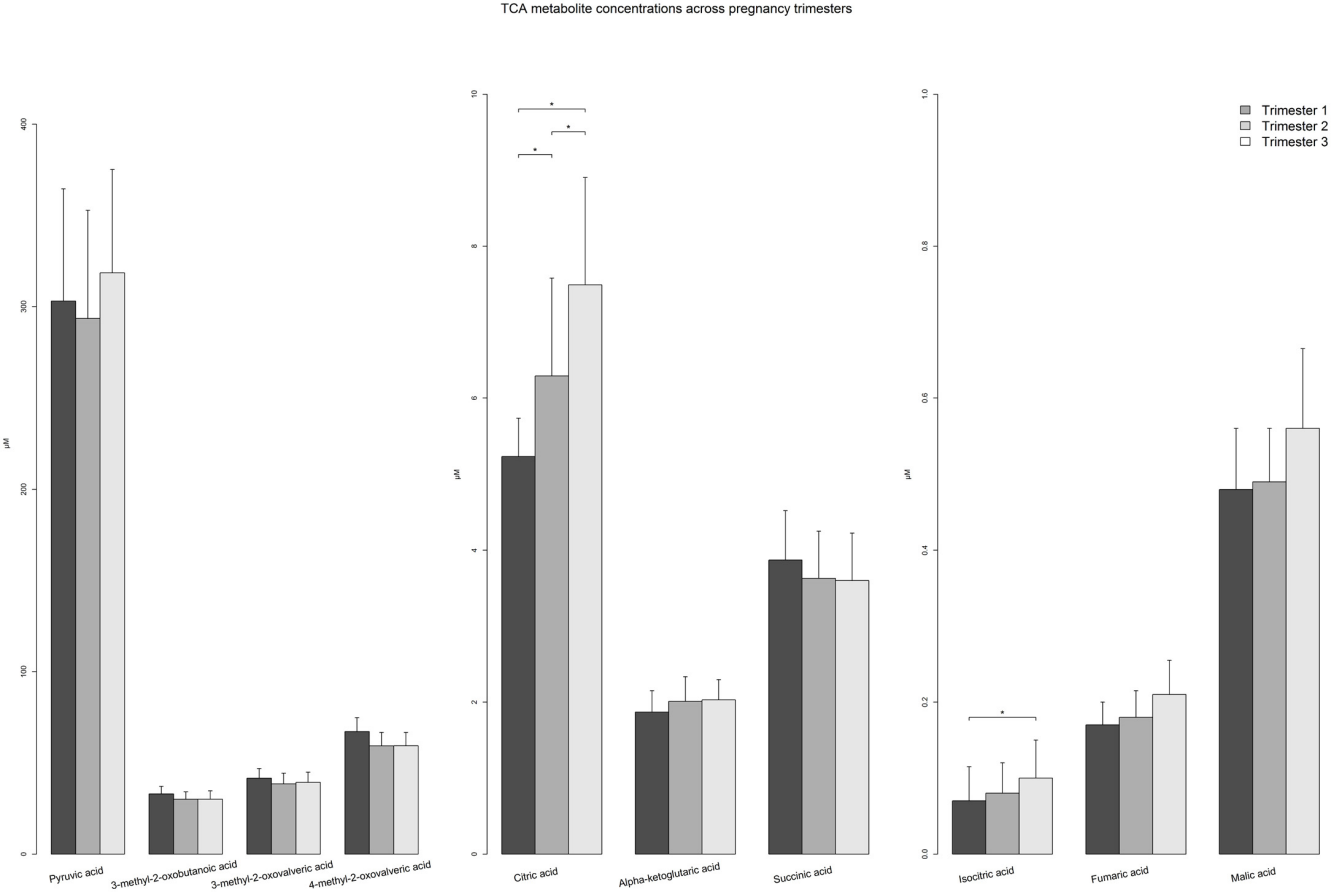
Barplot comparing plasma concentrations of tricarboxylic acid cycle intermediates and keto acids across trimesters among pregnant women. Median (+ Interquartile range/2) was plotted * p-value < 0.00017, p-value was calculated by Mann-Whitney U Test between trimesters.

The subsequent sections describe differences in plasma metabolite concentrations and metabolic ratios between Hispanic and non-Hispanic women and changes in these variables across pregnancy among the total population and stratified by ethnic group. For AA, NEFA, metabolic ratios, and TCA intermediates plus keto-acids, differences in median values between trimesters are visually presented in Figs 1, 2, 3 and 4, respectively (data presented in Tables A-D in S1 File). Data for polar lipids are presented in Table 2.

**Table 2:** Total population and ethnic-specific plasma Carn, Lyso-PL, PC.aa, PC.ae and SM concentrations in μmol/L within each trimester and comparison of median values between trimesters.

		Total Population (N = 160)		Hispanic Subjects (N = 68)		Non-Hispanic Subjects (N = 91)		Within-trimester Comparison^†^	Between-trimester Comparison^‡^			
Metabolite	Trimester	Median	IQR	Median	IQR	Median	IQR	p-value	Pair	Total population P-value	Hispanic subjects P-value	Non-Hispanic subjects P-value
Hexosen	T1	5538.67	1528.65	5715.43	1420.53	5294.02	1623.63	0.066	T2-T1	0.005	0.068	0.043
	T2	5303.97	1775.26	5560.85	1569.46	5193.43	1865.10	0.066	T3-T2	0.018	0.059	0.103
	T3	5612.92	1791.11	5896.33	1377.64	5408.94	1436.47	0.013	T3-T1	0.646	0.901	0.701
Free Carnitine	T1	26.55	9.89	26.55	9.55	27.09	11.65	0.817	T2-T1	7.080E-20	2.200E-09	7.830E-12
	T2	21.24	7.82	21.41	7.40	21.24	8.05	0.975	T3-T2	4.122E-15	1.000E-06	9.730E-10
	T3	18.19	6.32	18.63	6.54	18.07	5.95	0.687	T3-T1	2.513E-22	2.280E-10	2.230E-13
Carn.a.C10.1	T1	0.42	0.23	0.41	0.21	0.43	0.29	0.734	T2-T1	1.444E-07	8.500E-05	3.200E-04
	T2	0.34	0.22	0.34	0.17	0.35	0.24	0.722	T3-T2	0.008	0.070	0.072
	T3	0.30	0.17	0.27	0.16	0.34	0.18	0.073	T3-T1	2.688E-11	3.000E-06	3.000E-06
Carn.a.C12.0	T1	0.11	0.10	0.10	0.09	0.12	0.12	0.438	T2-T1	1.100E-05	0.002	0.001
	T2	0.09	0.07	0.10	0.07	0.09	0.07	0.548	T3-T2	0.285	0.165	0.823
	T3	0.09	0.07	0.08	0.05	0.10	0.06	0.058	T3-T1	6.090E-08	1.900E-05	7.800E-05
Carn.a.C12.1	T1	0.34	0.22	0.34	0.17	0.37	0.29	0.751	T2-T1	3.444E-07	2.400E-05	0.002
	T2	0.27	0.19	0.26	0.15	0.29	0.24	0.230	T3-T2	0.270	0.659	0.306
	T3	0.26	0.18	0.23	0.16	0.28	0.23	0.047	T3-T1	6.003E-08	2.600E-05	0.001
Carn.a.C14.0	T1	0.04	0.02	0.03	0.02	0.04	0.02	0.943	T2-T1	6.000E-06	1.33E-04	0.005
	T2	0.03	0.02	0.03	0.02	0.03	0.02	0.741	T3-T2	0.026	0.168	0.092
	T3	0.04	0.02	0.04	0.02	0.04	0.02	0.401	T3-T1	0.111	0.151	0.392
Carn.a.C14.1	T1	0.11	0.05	0.10	0.06	0.11	0.04	0.548	T2-T1	0.051	0.036	0.363
	T2	0.10	0.04	0.10	0.03	0.10	0.05	0.387	T3-T2	0.647	0.193	0.884
	T3	0.11	0.04	0.10	0.04	0.11	0.04	0.803	T3-T1	0.065	0.131	0.276
Carn.a.C14.2	T1	0.03	0.02	0.03	0.02	0.03	0.02	0.978	T2-T1	0.003	0.029	0.037
	T2	0.03	0.02	0.03	0.01	0.03	0.02	0.656	T3-T2	0.492	0.959	0.384
	T3	0.03	0.01	0.03	0.01	0.03	0.01	0.917	T3-T1	0.005	0.018	0.081
Carn.a.C16.0	T1	0.07	0.04	0.07	0.03	0.07	0.04	0.916	T2-T1	0.003	0.299	0.003
	T2	0.07	0.03	0.07	0.03	0.07	0.03	0.316	T3-T2	0.529	0.038	0.487
	T3	0.07	0.03	0.07	0.03	0.07	0.03	0.298	T3-T1	4.310E-04	0.002	0.055
Carn.a.C18.0	T1	0.03	0.02	0.03	0.02	0.03	0.02	0.541	T2-T1	0.780	0.364	0.267
	T2	0.03	0.02	0.03	0.02	0.03	0.02	0.896	T3-T2	0.056	0.168	0.153
	T3	0.03	0.02	0.03	0.02	0.03	0.02	0.605	T3-T1	0.078	0.420	0.098
Carn.a.C18.1	T1	0.11	0.06	0.12	0.07	0.11	0.05	0.754	T2-T1	0.036	0.043	0.228
	T2	0.11	0.05	0.10	0.05	0.11	0.05	0.444	T3-T2	0.634	0.216	0.755
	T3	0.11	0.05	0.10	0.05	0.11	0.04	0.011	T3-T1	0.065	0.006	0.911
Carn.a.C18.2	T1	0.09	0.06	0.08	0.05	0.09	0.05	0.462	T2-T1	0.012	0.397	0.08
	T2	0.08	0.04	0.09	0.04	0.08	0.04	0.224	T3-T2	0.281	0.066	0.888
	T3	0.08	0.04	0.07	0.04	0.08	0.04	0.222	T3-T1	0.005	0.014	0.096
Carn.a.C2.0	T1	5.30	2.91	5.52	2.73	5.25	3.19	0.894	T2-T1	9.992E-14	1.000E-06	3.452E-08
	T2	4.33	2.32	4.55	2.26	4.28	2.39	0.243	T3-T2	9.000E-05	0.002	0.011
	T3	3.93	1.79	3.68	2.11	4.01	1.67	0.689	T3-T1	7.664E-18	1.004E-08	1.770E-10
Carn.a.C3.0	T1	0.29	0.13	0.29	0.12	0.29	0.15	0.962	T2-T1	2.450E-09	0.001	1.000E-06
	T2	0.25	0.11	0.26	0.10	0.24	0.12	0.192	T3-T2	0.001	0.019	0.018
	T3	0.23	0.11	0.24	0.12	0.21	0.10	0.158	T3-T1	1.924E-16	5.675E-08	8.290E-10
Carn.a.C4.0	T1	0.19	0.10	0.18	0.09	0.20	0.11	0.139	T2-T1	2.440E-04	0.085	0.001
	T2	0.18	0.10	0.17	0.09	0.19	0.11	0.436	T3-T2	0.136	0.402	0.240
	T3	0.16	0.10	0.16	0.08	0.16	0.08	0.226	T3-T1	2.320E-04	0.012	0.008
Carn.a.C8.1	T1	0.11	0.07	0.11	0.06	0.11	0.08	0.906	T2-T1	0.001	0.008	0.047
	T2	0.09	0.06	0.09	0.06	0.09	0.07	0.573	T3-T2	0.477	0.768	0.291
	T3	0.09	0.05	0.09	0.05	0.09	0.06	0.759	T3-T1	0.001	0.014	0.028
Carn.a.C9.0	T1	0.02	0.01	0.02	0.01	0.02	0.01	0.003	T2-T1	0.782	0.808	0.732
	T2	0.02	0.01	0.01	0.01	0.02	0.02	0.023	T3-T2	0.330	0.815	0.154
	T3	0.02	0.01	0.02	0.01	0.02	0.01	0.026	T3-T1	0.020	0.538	0.014
Sum of short-chain Carn (N = 3)	T1	5.68	2.65	5.72	2.81	5.69	2.69	0.546	T2-T1	8.456E-10	9.500E-05	3.000E-06
	T2	4.25	1.86	4.50	2.16	4.04	1.69	0.098	T3-T2	0.016	0.029	0.244
	T3	4.14	1.65	4.14	2.01	4.23	1.55	0.776	T3-T1	1.948E-11	1.900E-05	3.476E-07
Sum of medium-chain Carn (N = 5)	T1	1.03	0.06	1.00	0.51	1.05	0.64	0.585	T2-T1	6.171E-08	3.800E-05	2.460E-04
	T2	0.84	0.52	0.84	0.45	0.85	0.60	0.319	T3-T2	0.057	0.359	0.108
	T3	0.77	0.44	0.73	0.41	0.83	0.49	0.054	T3-T1	6.704E-10	9.000E-06	3.200E-05
Sum of long-chain Carn (N = 7)	T1	0.45	0.22	0.48	0.22	0.44	0.19	0.581	T2-T1	0.025	0.051	0.176
	T2	0.45	0.20	0.47	0.19	0.43	0.18	0.406	T3-T2	0.749	0.722	0.424
	T3	0.46	0.19	0.45	0.17	0.47	0.23	0.591	T3-T1	0.250	0.067	0.946
Sum of all Lyso-PL (N = 17)	T1	115.82	45.61	114.64	36.38	122.33	55.18	0.188	T2-T1	1.66E-09	1.657E-09	1.657E-09
	T2	104.00	37.02	103.23	38.64	104.87	36.47	0.910	T3-T2	2.41E-04	2.410E-04	2.410E-04
	T3	97.70	36.47	93.35	34.81	99.87	36.39	0.213	T3-T1	3.21E-12	3.208E-12	3.208E-12
Sum of all PCaa (N = 39)	T1	2139.44	708.73	2228.00	701.31	2066.33	685.23	0.099	T2-T1	1.68E-11	1.684E-11	1.684E-11
	T2	2368.87	801.65	2467.51	896.88	2333.21	721.34	0.142	T3-T2	4.06E-11	4.063E-11	4.063E-11
	T3	2657.32	805.97	2668.68	839.64	2624.26	714.32	0.442	T3-T1	2.15E-18	2.155E-18	2.155E-08
Sum of all PCae (N = 37)	T1	232.84	74.88	232.84	76.93	232.00	70.53	0.748	T2-T1	3.00E-06	3.000E-06	3.000E-06
	T2	256.96	83.06	256.84	101.71	259.78	77.20	0.966	T3-T2	0.174	0.174	0.174
	T3	262.24	82.07	259.54	84.91	262.46	80.40	0.930	T3-T1	1.87E-08	1.874E-08	1.874E-04
Sum of all SM (N = 54)	T1	528.91	105.31	554.11	108.36	499.10	142.01	0.043	T2-T1	5.97E-12	6.000E-12	5.965E-12
	T2	596.28	137.36	612.32	180.53	571.19	166.82	0.052	T3-T2	2.50E-12	3.000E-12	2.504E-12
	T3	664.80	150.15	695.88	226.64	632.79	166.02	0.058	T3-T1	5.89E-18	6.000E-18	5.893E-18

Carn, acylcarnitines; IQR, interquartile range; Lyso-PL, lysophospholipid; PCaa, diacyl-phosphotidylcholine; PCae, acyl-alkyl-phosphotidylcholine; SM, sphingomyelin.

^†^P-values represent difference in median metabolite values within a given trimester between ethnic groups, calculated by Mann-Whitney U test.

^‡^P-values represent difference in median metabolite values between paired trimesters, calculated by Wilcoxon Rank test separately for total population, Hispanic subjects and non-Hispanic subjects. Significance set at p<0.05. Bonferroni correction for multiple comparisons (N = 97*3 timepoints = 291) implies values are statistically significant where p <0.00017 (1.7E-4).

### Amino acids

Various significant changes in median AA levels are observed across pregnancy (Fig 1). The sum of BCAA significantly decreases as gestation progresses, driven by changes in leucine and valine, although the overall decrease is more pronounced among non-Hispanic women (Table A in S1 File). While the sum of all essential AA does not reveal any significant changes across trimesters, when the BCAA are excluded from this group, the sum of the remaining essential AA significantly increases from first to third trimester. Closer inspection of the individual non-branched chain essential AA reveals that threonine alone is driving this group increase, as it steeply rises by 30 μmol/L between the first and third trimester. Meanwhile, phenylalanine and tryptophan decrease across pregnancy, while no significant change is observed in methionine levels. Regarding non-essential AA, there is a general trend towards decreasing levels across trimesters, with the exception of glutamic acid which is significantly increased in the third trimester compared to both the first and second trimester levels. There is a trend towards higher levels of some non-essential AA throughout pregnancy (asparagine, aspartic acid, citrulline, glycine, glutamine) among non-Hispanic compared to Hispanic women.

While not definitely an amino acid due to the absence of any carboxyl group, concentrations of the sulphur-containing organic acid taurine are observed to significantly decrease in the third trimester compared to the first among all subjects.

### Non-esterified fatty acids

For the saturated and monounsaturated NEFA metabolites, no clear changes over time are observed across pregnancy (Fig 2), and any differences in concentrations between trimesters are non-significant after correction for multiple testing. Among the polyunsaturated NEFA, it is noted that the levels of C18:2 and C18:3 do not significantly change between trimesters among the total population, although they are higher among Hispanic women in early pregnancy (Table B in S1 File). Although the metabolomic analysis technique cannot differentiate the location of double bonds, it can be assumed that these particular NEFA represent the omega-3 and omega-6 essential fatty acids, α-linolenic acid and linoleic acid, respectively. Meanwhile, several long-chain polyunsaturated fatty acids (LC-PUFA) are observed to significantly decrease from first to second trimester and remain relatively stable between second and third trimesters. Ethnic-specific differences are observed for the conditionally essential LC-PUFA docosahexanoic acid (DHA, C22:6n-3) and eicosapentanoic acid (EPA, C20:5n-3), which are biosynthesized from C18:3n-3, such that Hispanic women have lower concentrations than non-Hispanic women and the decrease in DHA with advancing gestation among the total population is predominantly driven by a decrease in the Hispanic group.

### Polar lipids

Longitudinal analysis of Carn levels across pregnancy reveals rather specific changes (Table 2). Both free carnitine and acetylcarnitine (Carn.a.C2.0), demonstrate a significant decrease across trimesters, with a similar trend observed among both ethnic groups. Levels of the other short-chain Carn also significantly decrease although the magnitude of their changes is not as steep. Meanwhile, first trimester levels of a few medium-chain Carn are significantly higher than levels in later pregnancy, while long-chain Carn levels remain almost completely stable throughout gestation. Among the remaining polar lipids, lyso-PL significantly decreased across trimesters while the sum of all PCaa, PCae and SM groups each significantly increased in their plasma concentrations with advancing gestation among all women regardless of ethnicity.

Among the ratios calculated as indicators of metabolic processes (Fig 3 and Table C in S1 File), each of the 5 ratios used as a possible indicator of CPT-1 rate increased significantly between trimesters, reflecting the decreasing levels of free carnitine which is used as the denominator for each ratio. Conversely, the 5 ratios used to indicate β-oxidation rate are each significantly higher in the first trimester compared to levels in trimesters 2 and 3. This reflects the progressive decrease in Carn.a.C2 across pregnancy, which is the numerator for each β-oxidation ratio.

### Metabolites involved in amino acids and fatty acid degradation

Several intermediate metabolites of the TCA cycle, namely citric acid, isocitric acid, α-ketoglutaric acid, fumaric acid and malic acid, are significantly increased in concentration in later pregnancy compared to the first trimester (Fig 4). In particular, citric acid, the first product of acetyl-CoA metabolism, steadily rises across trimesters in the total population and the individual ethnic groups. However, pyruvic acid, a common substrate for generation of acetyl-CoA molecules through glucose oxidation does not significantly change in concentration throughout pregnancy. Meanwhile, the keto-acids produced from the first stage of BCAA oxidation demonstrate either minimal change (3-methyl-2-oxobutanoic acid and 3-methyl-2-oxovalveric acid) or a decrease in concentration (4-methyl-2-oxovalveric acid) across gestation. Beta-hydroxy-butyric acid, a product of ketogenesis, significantly rises across gestation in the total population, particularly in the third trimester. Interestingly among non-Hispanic subjects, this keto-body initially decreases from first to second trimester but then recovers in the third trimester to match the elevated levels of the Hispanic women (Table D in S1 File).

## Discussion

To our knowledge, this is the first study to comprehensively describe the longitudinal changes in maternal metabolites among healthy pregnant women using targeted metabolomic profiling. To date, the nutritional application of metabolomics in pregnancy has largely focused on those with glucose intolerance in comparison to women with normal glucose tolerance [15–17] or the correlation of protein metabolites with fetal growth and intra-uterine growth retardation [18–19]. While recent cross-sectional studies have provided initial insight to metabolomic profiles in healthy pregnancy [7–9], the longitudinal design, broad range of targeted metabolic products and intermediates analyzed, and consideration of ethnic influences in the present study provide critical advancement to the characterization of metabolism in pregnancy through a deeper understanding of changes in the maternal metabolome with advancing gestation and inter-individual differences by ethnicity.

### Amino acids and protein metabolism

The decrease in several maternal plasma AA levels across trimesters is consistent with the concept of placental AA uptake and fetal transfer for protein synthesis [18]. Fetal plasma AA concentrations have been reported to be significantly higher than maternal levels [15,20]. Although, we do not have non-pregnant AA levels as a comparison, maternal hypoaminoacidaemia in pregnancy is known to occur in the fasted state from as early as the first trimester and to persist throughout pregnancy [1]. Several AAs are also potential substrates for gluconeogenesis in the fasted state, although it is generally accepted that their use in these processes is diminished during gestation as protein anabolism rather than catabolism is favored [21]. Reduced plasma concentrations of gluconeogenic AAs during pregnancy is thought to contribute to the accelerated occurrence of fasting hypoglycaemia compared to non-pregnant women [22,23].

The variations in the changing patterns across trimesters of individual AAs from our data highlight the unique nature of each AA in pregnancy and metabolism. While the sum of non-essential AAs does not differ significantly between trimesters, a significant decreasing pattern in concentrations of arginine, glycine, serine, tyrosine and cysteine is observed. Reduced concentrations of these metabolites may reflect reduced rate of biosynthesis. For example, the plasma concentration of the essential AA phenylalanine decreases from the first trimester, probably due to its conservation for nitrogen accretion and tissue synthesis [24] thereby reducing its availability for its alternative role in tyrosine biosynthesis [25]. In contrast, glutamic acid significantly increases in the third trimester which is similar to data reported by Di Giulio et al [19]. We hypothesize that this increase may be related to its reduced conversion to glutamine secondary to a reduced α-amino nitrogen pool [21], which is reflected in the stable glutamine levels throughout gestation in our data. The significant increase in the essential AA threonine with advancing gestation was previously reported [19]. Rees et al [26] studied threonine metabolism in the pregnant rat and concluded that dietary protein induced increases in serine-threonine dehydratase activity favored conservation of this AA rather than its oxidation.

Although we report an overall decrease in the sum of BCAA across pregnancy trimesters, it is specifically leucine and valine which significantly decrease, while plasma isoleucine concentrations remain more stable. BCAA are a sub-group of essential AA which may undergo transamination to generate nitrogen for synthesis of non-essential AA such as glutamine and alanine [27]. Maternal transamination and urea synthesis during pregnancy has been shown to decrease [21], however, which is supported by the decreased or unchanged concentrations of α-keto-acids in the current data as these are the initial products of BCAA metabolism. On the other hand, high activity of the transamination enzymes, branched-chain aminotransferases, have been found in human placental tissue [28], providing evidence for placental BCAA uptake where they are transaminated in order to sustain the high placental nitrogen demand that is used for several metabolic functions including glutamate synthesis [28]. This may also account for the decreasing pattern of BCAA despite the normal, progressive insulin resistance that occurs during pregnancy [3], which would conflict with evidence from non-pregnant human studies reporting higher concentrations of BCAA associated with insulin resistance [29–31].

An alternative potential fate for the AA is their use as substrates for ketogenesis in order to generate ketone bodies as a source of fuel while maternal glucose supply is low. Plasma samples for the present study were all collected from women following an overnight fast and as previously alluded to, a more rapid progression towards hypoglycaemia occurs in pregnant women in the fasting state, a concept referred to as ‘accelerated starvation’ [23]. In response to this, initiation of ketogenesis to subsidize maternal energy demands has been reported to occur more rapidly in pregnancy compared to non-pregnant women [3] and indeed, the increased concentration of β-hydroxybutyrate with advancing gestation in this cohort suggests that ketogenesis is occurring in the fasted state. However, NEFA also serve as efficient ketogenic substrates and following the concept of protein accretion in pregnancy, it would be in agreement with existing evidence surrounding maternal metabolism that NEFA would be the ketogenic substrates of preference in these fasted subjects.

The decrease in plasma taurine concentrations is likely attributed to uptake by placental and maternal tissues [32] as adequate reserves of this non-protein AA are critical for fetal growth and neurodevelopment in the newborn, and reduced urinary excretion during pregnancy has been previously demonstrated [33].

### Non-esterified fatty acids and lipid metabolism

The normal fat deposition in maternal adipose stores associated with early pregnancy is followed by lipolysis in late gestation as insulin resistance increases, with lipolytic activity being further enhanced under fasting conditions [34]. Lipolysis of triacylglycerol in adipose tissue releases glycerol and NEFA into the maternal circulation where they are transported to peripheral tissue and used for energy provision by beta-oxidation and ketogenesis in the fasted state [4]. Furthermore, NEFA are taken up by the liver for re-synthesis of triacylglycerols. Our data does not indicate any increase in plasma NEFA concentrations, however, and where significant differences in levels do occur between trimesters for LC-PUFA, they relate to reduced concentrations in later pregnancy compared to the first trimester.

A fetal supply of LC-PUFA is essential for fetal nervous system development [35]. While placental uptake of NEFA is possible, evidence suggests that LC-PUFA are preferentially taken up from the maternal circulation in the form of triacylglycerols present in lipoproteins [36,37]. In addition, FA may be delivered by phospholipids to the placenta, as evidenced by the high placental phospholipase activity [35]. Thus, the observed reduction in maternal plasma LC-PUFA in later pregnancy in our data may reflect their conversion to triacylglycerols and/or phospholipids and subsequent placental uptake. Although we do not have available data on maternal triacylglycerol concentrations to support this hypothesis, the increasing maternal plasma concentrations of phosphatidylcholines and sphingomyleins in our data likely indicate the enhanced role of phospholipids in NEFA transport to the fetus as pregnancy progresses. Interestingly, the lower baseline levels of DHA and EPA among Hispanic women compared to non-Hispanic women, despite having higher levels of their precursor α-linolenic acid, would suggest that non-Hispanic women have a higher dietary intake of these conditionally essential LC-PUFA which are predominantly sourced from oily fish or nutritional supplements. Indeed, suboptimal dietary intake of EPA and DHA has been reported among pregnant women of Hispanic ethnicity living in the US [38], and the plasma levels in our data confirm that this is a nutritional concern which may affect fetal neurological and retinal development [39].

A further explanation for the absence of any increase in plasma NEFA levels in our data could be enhanced delivery of NEFA to the maternal liver for β-oxidation to acetyl-CoA and ketone body synthesis. While the concentrations of several TCA cycle intermediates are seen to increase, pyruvic acid levels remain stable, thus supporting the concept that fatty acids rather that carbohydrate are being oxidized to generate acetyl-CoA as pregnancy progresses, at least in the fasted state in this cohort. The concomitant rise in β-hydroxybutyrate also suggests that NEFA are undergoing ketogenesis as an alternative method of energy production. Fasting increases adipose tissue lipolysis in late gestation in order to generate a maternal energy supply when glucose availability is low [4] and the ‘accelerated starvation’ phenomenon of pregnancy induces ketogenesis after only slightly prolonged periods of fasting [2,37]. In addition to serving as a maternal energy source, ketone bodies, such as β-hydroxybutyrate, are efficiently transferred across the placenta to the fetus to serve as fuel and substrates for brain lipid synthesis [37]. The ethnic-specific analysis of our data suggests that this process takes significant effect only in the third trimester among non-Hispanic women. Meanwhile, the steady rise in β-hydroxybutyrate throughout pregnancy among Hispanic women could suggest more efficient metabolic adaptation to fasting earlier in pregnancy, or could be an indicator of alternative dietary patterns in the second trimester, e.g. not consuming food late in the evening and subsequently undergoing a longer period of fasting. Furthermore, longitudinal changes in free carnitine and acylcarnitines provide further insight to the metabolic pathways being utilized throughout gestation.

### Free carnitine, acylcarnitines and metabolic ratios

The significant decline in free carnitine and short-chain acylcarnitines across trimesters in our subjects is in agreement with recent non-targeted metabolomic data from the study by Luan et al. [7], which also measured these metabolites among different samples of pregnant women at various stages in gestation. Earlier studies have also reported a decline in plasma free carnitine and increased renal excretion of acylcarnitines during pregnancy [40,41]. Carnitine is primarily sourced from dietary intakes of animal protein foods (meat, fish and dairy), but biosynthesis contributes approximately 25% to circulating carnitine levels [42].

Carnitine is an essential compound required for fatty acid metabolism, which occurs in the mitochondria of cells. As NEFA are transported across cell membranes, they are rapidly converted to acyl-Coenzyme A compounds (acyl-CoAs) by fatty acid transport proteins [43]. As the longer chain acyl-CoAs are impermeable to the inner mitochondrial membrane they must be converted to long-chain acylcarnitines by CPT1, and are re-converted back to acyl-CoA by CPT2 once inside the mitochondria [44]. Acyl-CoAs then undergo the β-oxidation cycle which generates acetyl-CoA molecules. In the fed state, fatty acid oxidation is completed through transfer of acetyl-CoA to the TCA cycle to generate substrates for the ATP-providing electron transport chain [43]. Under prolonged fasting conditions, however, the TCA cycle is inhibited and acetyl-CoA is used to produce ketone bodies as an alternative fuel source through the process of ketogenesis [45]. Utilization of acetyl-CoA in ketogenesis decreases the concentrations of malonyl-CoA, a potent inhibitor of the CPT1 enzyme [42]. This facilitates enhanced fatty acid metabolism by increasing the rate of acyl-CoA transfer into mitochondria.

This process of fatty acid metabolism also applies to healthy human pregnancy, particularly in the third trimester when maternal NEFA supply is enhanced with increased lipolytic activity. The main difference in pregnancy is that the switch from fatty acid oxidation to ketogenesis appears to occur more rapidly under fasting conditions than in the non-pregnant state, probably due to increased fetal demands [2]. As previously mentioned, the increased concentrations of both TCA cycle intermediates and ketone bodies detected in this study suggests enhanced oxidative and non-oxidative metabolic activity with advancing gestation. Regardless of the final metabolic fate however, it seems plausible that the reduced free carnitine concentrations reported in our data among pregnant women following an overnight fast reflect the increased use of this compound for acyl-CoA transport into mitochondria to provide the necessary substrates for energy production. This is also reflected in the increased ratios for estimating CPT1 activity with advancing gestation, and may be associated with the decreased concentrations of long-chain NEFA in the second and third trimesters as these are converted to long-chain acyl-CoAs following transport into cells, the primary substrates for CPT1 activity [43].

However, carnitine is also required by the developing fetus for both fetal maturation and fatty acid oxidation in the placental-fetal unit [46]. This supply is thought to be primarily met through placental carnitine uptake from the maternal circulation [47], which therefore may also contribute to the reduction in maternal plasma carnitine levels throughout pregnancy. Alternatively, there is evidence that the human placenta and fetus are capable of complete carnitine biosynthesis [48]. This mechanism of carnitine production is thought to be particularly important under conditions of poor maternal carnitine supply, such as in the case of a poor vegan diet [49]. However, the impact of the maternal fasting state in pregnancy on carnitine supply to the fetus has not been investigated to our knowledge.

Although we do not have measures of urinary acylcarnitine concentrations, previous reports of increased urinary excretion of acylcarnitines across pregnancy [40,41,50] may explain the reduced plasma concentrations of certain carnitine esters among our subjects. The most pronounced decrease in plasma acylcarnitine concentrations was observed for acety-L-carnitine, which is also a precursor for acetyl-CoA. The metabolomic evidence presented for increasing rates of TCA cycle activity and fasting-induced ketogenesis with advancing gestation among this population would increase the demand for acetyl-CoA supply, thus potentially contributing to the reduced circulating acetyl-L-carnitine levels.

### Strengths, Limitations and Future Directions

Our study is strengthened by a relatively large sample size and use of a targeted metobolomic technique to longitudinally analyze a broad range of distinct nutrient metabolites of interest across gestation. The inclusion of intermediates of metabolic processes also facilitated an in-depth interpretation of the metabolic fate of various nutrients at different stages of gestation, while stratification of analyses by the dominant ethnic groups offers insight to the potential for inter-individual variation in prenatal metabolism and/or dietary habits that influence the metabolome and could be addressed through public health measures. The absence of other parameters, such as plasma triglycerides, urinary carnitine esters or tissue protein and lipid concentrations, however, limits our complete interpretation of metabolomic profiles in these pregnant subjects. Also, as blood samples were collected from subjects in the fasted state, these results do not reflect potential changes in the maternal metabolome across gestation that may occur post-prandial.

This data demonstrates the value of metabolomic research in evaluating whole body metabolism, particularly in pregnancy which represents a complexity of metabolic alterations from the non-pregnant state and with advancing gestation. We provide a foundation for further metabolomic profiling studies in pregnancy to advance our understanding of maternal and fetal metabolism during this critical life-stage and highlight the importance of considering ethnic or other phenotypic diversity which may impact on the maternal metabolome. Indeed, subtle differences were observed for several metabolites between Hispanic and non-Hispanic women in this study including some non-essential AA, essential long-chain NEFA and ketone bodies, all of which may have implications for maternal nutritional status and fetal metabolism. Knowledge of the flux of nutrients and other metabolites between the maternal and placental-fetal unit is in its infancy and further metabolomic research in this field may advance our understanding of the effects of maternal biochemistry, physiology and lifestyle behaviors on fetal programming and infant outcomes. Thus, there is enormous scope for future studies to utilize metabolomic techniques to understand alterations in maternal metabolism in response to different maternal characteristics, dietary intakes, body compositions and hormonal alterations across pregnancy, as well as metabolomic associations with fetal and neonatal outcomes.

## Supporting Information

S1 File Total population and ethnic-specific plasma concentrations of amino acids (Table A), NEFA (Table B), metabolic ratios (Table C) and ketogenesis and TCA intermediates (Table D), within each trimester and comparison of median values between trimesters.
(DOCX)

## Abbreviations

AA: Amino acids
BCAA: Branched chain amino acids
Carn: Acylcarnitines
CPT: Carnitine palmitoyltransferase
DHA: Docosahexaenoic acid
EDTA: Ethylenediamine tetra-acetic acid
ESI: Electrospray ionization
FA: Fatty acids
FIA: Flow injection analysis
HPLC: High performance liquid chromatography
IQR: Interquartile range
LC: Liquid chromatography
LC-PUFA: Long-chain polyunsaturated fatty acids
Lyso-PL: Lysophospholipids
MS: Mass spectrometry
MRM: Multiple Reaction Monitoring
NEFA: Non-esterified fatty acids
PCaa: Diacyl- phosphatidylcholines
PCae: Acyl-alkyl-phosphatidylcholines
QC: Quality control
SD: Standard deviation
SM: Sphingomyelins
TCA: Tricarboxylic acid
