# Supplementary material for: Longitudinal Metabolomic Profiling of Amino Acids and Lipids across Healthy Pregnancy
Source: PLoS One. 2015 Dec 30;10(12):e0145794. doi: 10.1371/journal.pone.0145794 (PMC4699222; doi:10.1371/journal.pone.0145794)
Supplement: S1 File — Total population and ethnic-specific plasma concentrations of amino acids (Table A), NEFA (Table B), metabolic ratios (Table C) and ketogenesis and TCA intermediates (Table D), within each trimester and comparison of median values between trimesters. (DOCX) [file pone.0145794.s001.docx]

**Table A: Total population and ethnic-specific plasma amino acid concentrations (µmol/L) within each trimester and comparison of median values between trimesters**

|  |  | **Total Population (N=160)** | | **Hispanic Subjects (N=68)** | | **Non-Hispanic Subjects (N=91)** | | **Within-trimester Comparison^†^** | **Between-trimester Comparison^‡^** | | | |
| --- | --- | --- | --- | --- | --- | --- | --- | --- | --- | --- | --- | --- |
| **Amino acid** | **Trimester** | **Median** | **IQR** | **Median** | **IQR** | **Median** | **IQR** | **P-value** | **Pair** | **Total population P-value** | **Hispanic subjects P-value** | **Non-Hispanic subjects P-value** |
| Alanine | T1 | 252.00 | 80.00 | 250.00 | 90.00 | 253.00 | 83.00 | 0.507 | T2-T1 | 0.325 | 0.666 | 0.066 |
|  | T2 | 263.00 | 76.50 | 262.00 | 81.00 | 265.00 | 76.00 | 0.355 | T3-T2 | 0.031 | 0.249 | 0.049 |
|  | T3 | 261.50 | 73.25 | 263.00 | 65.00 | 262.00 | 86.50 | 0.422 | T3-T1 | 0.090 | 0.884 | 0.024 |
| Arginine | T1 | 27.00 | 11.45 | 26.30 | 11.50 | 27.65 | 10.83 | 0.841 | T2-T1 | 2.000E-06 | 0.021 | 1.300E-05 |
|  | T2 | 24.10 | 9.10 | 25.40 | 9.70 | 23.20 | 9.00 | 0.057 | T3-T2 | 0.001 | 0.051 | 0.008 |
|  | T3 | 23.35 | 10.88 | 23.70 | 10.40 | 22.10 | 11.50 | 0.209 | T3-T1 | 2.543E-10 | 9.900E-05 | 4.204E-07 |
| Asparagine | T1 | 33.10 | 7.90 | 31.10 | 8.40 | 34.20 | 7.90 | 0.003 | T2-T1 | 1.000E-06 | 2.160E-04 | 0.001 |
|  | T2 | 36.00 | 9.70 | 34.00 | 8.55 | 37.60 | 7.80 | 0.008 | T3-T2 | 0.001 | 0.008 | 0.041 |
|  | T3 | 33.85 | 8.53 | 32.10 | 8.60 | 34.95 | 9.95 | 0.002 | T3-T1 | 0.222 | 0.418 | 0.289 |
| Aspartic acid | T1 | 7.37 | 2.44 | 6.79 | 2.30 | 7.58 | 2.41 | 0.008 | T2-T1 | 0.034 | 0.072 | 0.193 |
|  | T2 | 7.39 | 2.39 | 7.10 | 2.41 | 7.51 | 2.62 | 0.062 | T3-T2 | 0.090 | 0.289 | 0.199 |
|  | T3 | 7.64 | 2.67 | 7.29 | 2.20 | 7.83 | 3.15 | 0.015 | T3-T1 | 0.002 | 0.093 | 0.009 |
| Citrulline | T1 | 12.40 | 4.60 | 11.50 | 4.77 | 13.10 | 4.45 | 0.002 | T2-T1 | 0.026 | 0.291 | 0.064 |
|  | T2 | 12.40 | 3.85 | 11.40 | 3.35 | 13.00 | 4.80 | 0.013 | T3-T2 | 0.332 | 0.861 | 0.244 |
|  | T3 | 12.15 | 4.03 | 11.00 | 4.57 | 12.80 | 3.43 | 0.008 | T3-T1 | 0.011 | 0.473 | 0.005 |
| Glutamine | T1 | 397.00 | 81.00 | 378.00 | 74.50 | 413.00 | 86.50 | 0.011 | T2-T1 | 0.954 | 0.639 | 0.517 |
|  | T2 | 398.00 | 80.00 | 380.00 | 71.50 | 408.00 | 70.00 | 0.001 | T3-T2 | 0.001 | 0.028 | 0.028 |
|  | T3 | 388.50 | 88.00 | 367.00 | 66.00 | 402.00 | 98.25 | 0.001 | T3-T1 | 0.008 | 0.004 | 0.436 |
| Glutamic acid | T1 | 54.90 | 14.95 | 54.70 | 14.45 | 54.90 | 14.93 | 0.590 | T2-T1 | 0.898 | 0.344 | 0.268 |
|  | T2 | 53.40 | 15.80 | 53.50 | 16.50 | 53.20 | 14.50 | 0.735 | T3-T2 | 1.096E-08 | 0.002 | 1.000E-06 |
|  | T3 | 60.15 | 19.03 | 60.20 | 19.20 | 59.95 | 18.05 | 0.504 | T3-T1 | 3.018E-08 | 1.540E-04 | 5.400E-05 |
| Glycine | T1 | 120.00 | 44.75 | 115.00 | 38.60 | 131.00 | 43.50 | 0.001 | T2-T1 | 1.310E-04 | 0.181 | 1.810E-04 |
|  | T2 | 116.00 | 39.50 | 106.50 | 36.58 | 123.00 | 37.00 | 0.002 | T3-T2 | 8.000E-05 | 0.065 | 0.001 |
|  | T3 | 112.00 | 40.95 | 103.00 | 33.40 | 117.00 | 45.93 | 0.012 | T3-T1 | 3.772E-09 | 4.960E-04 | 5.000E-06 |
| Isoleucine | T1 | 37.70 | 9.83 | 36.40 | 7.625 | 38.00 | 10.10 | 0.337 | T2-T1 | 0.106 | 0.274 | 0.275 |
|  | T2 | 36.30 | 9.60 | 35.90 | 8.50 | 37.90 | 10.50 | 0.191 | T3-T2 | 0.074 | 0.740 | 0.046 |
|  | T3 | 35.10 | 11.20 | 34.50 | 11.40 | 35.50 | 10.65 | 0.645 | T3-T1 | 0.002 | 0.176 | 0.006 |
| Leucine | T1 | 78.80 | 20.03 | 76.65 | 23.83 | 80.80 | 19.30 | 0.302 | T2-T1 | 6.700E-05 | 0.181 | 1.120E-04 |
|  | T2 | 74.20 | 20.25 | 75.75 | 18.80 | 74.00 | 20.80 | 0.883 | T3-T2 | 2.930E-04 | 0.078 | 0.001 |
|  | T3 | 71.75 | 19.83 | 73.20 | 20.00 | 70.45 | 19.45 | 0.431 | T3-T1 | 2.388E-09 | 0.002 | 4.092E-07 |
| Methionine | T1 | 15.00 | 3.45 | 14.95 | 2.38 | 15.05 | 4.23 | 0.593 | T2-T1 | 0.006 | 0.586 | 0.005 |
|  | T2 | 14.70 | 3.40 | 14.70 | 2.53 | 14.65 | 3.78 | 0.911 | T3-T2 | 0.893 | 0.347 | 0.428 |
|  | T3 | 14.60 | 3.50 | 14.80 | 3.00 | 14.50 | 3.73 | 0.381 | T3-T1 | 0.001 | 0.373 | 0.001 |
| Ornithine | T1 | 33.20 | 14.60 | 32.10 | 16.40 | 35.40 | 13.95 | 0.099 | T2-T1 | 0.013 | 0.052 | 0.135 |
|  | T2 | 30.60 | 11.35 | 29.00 | 9.33 | 32.00 | 14.20 | 0.002 | T3-T2 | 0.231 | 0.434 | 0.277 |
|  | T3 | 31.74 | 10.25 | 28.35 | 10.50 | 32.05 | 11.65 | 0.003 | T3-T1 | 4.720E-04 | 0.005 | 0.021 |
| Phenylalanine | T1 | 42.15 | 9.88 | 40.75 | 9.17 | 42.30 | 10.95 | 0.159 | T2-T1 | 1.890E-04 | 0.564 | 5.000E-05 |
|  | T2 | 39.90 | 8.80 | 39.60 | 8.85 | 40.50 | 8.40 | 0.893 | T3-T2 | 0.521 | 0.457 | 0.136 |
|  | T3 | 39.40 | 7.85 | 41.00 | 9.20 | 38.50 | 6.45 | 0.255 | T3-T1 | 1.030E-04 | 0.547 | 2.400E-05 |
| Proline | T1 | 118.00 | 50.20 | 123.00 | 45.70 | 112.00 | 53.15 | 0.405 | T2-T1 | 0.463 | 0.614 | 0.278 |
|  | T2 | 115.00 | 46.00 | 119.00 | 49.00 | 114.00 | 44.00 | 0.149 | T3-T2 | 0.064 | 0.093 | 0.432 |
|  | T3 | 119.50 | 41.93 | 123.00 | 39.00 | 114.00 | 44.33 | 0.030 | T3-T1 | 0.915 | 0.582 | 0.608 |
| Tryptophan | T1 | 44.55 | 8.85 | 43.30 | 8.00 | 45.60 | 9.53 | 0.322 | T2-T1 | 1.083E-08 | 0.003 | 9.762E-07 |
|  | T2 | 40.20 | 8.90 | 40.50 | 8.55 | 40.10 | 9.70 | 0.715 | T3-T2 | 3.555E-10 | 7.500E-05 | 2.000E-06 |
|  | T3 | 36.25 | 6.80 | 36.80 | 7.00 | 35.95 | 6.65 | 0.597 | T3-T1 | 4.840E-19 | 2.661E-08 | 4.995E-12 |
| Serine | T1 | 61.10 | 20.80 | 60.20 | 25.45 | 61.80 | 20.15 | 0.887 | T2-T1 | 2.498E-09 | 6.800E-05 | 9.000E-06 |
|  | T2 | 54.70 | 15.00 | 52.45 | 15.25 | 55.70 | 12.75 | 0.232 | T3-T2 | 0.481 | 0.271 | 0.043 |
|  | T3 | 52.60 | 15.35 | 51.80 | 16.60 | 52.60 | 15.35 | 0.902 | T3-T1 | 6.650E-12 | 4.600E-05 | 4.678E-08 |
| Threonine | T1 | 106.00 | 38.20 | 106.00 | 46.15 | 106.00 | 34.35 | 0.586 | T2-T1 | 5.143E-14 | 3.000E-06 | 6.819E-10 |
|  | T2 | 124.00 | 50.50 | 119.00 | 52.00 | 131.50 | 48.45 | 0.450 | T3-T2 | 5.000E-06 | 0.002 | 0.001 |
|  | T3 | 136.50 | 56.75 | 141.00 | 55.00 | 136.00 | 55.00 | 0.748 | T3-T1 | 3.910E-18 | 2.481E-09 | 1.766E-10 |
| Tyrosine | T1 | 35.40 | 9.60 | 35.80 | 8.10 | 35.00 | 10.60 | 0.781 | T2-T1 | 3.729E-08 | 0.004 | 3.000E-06 |
|  | T2 | 33.25 | 6.75 | 33.85 | 6.68 | 32.50 | 6.80 | 0.729 | T3-T2 | 0.315 | 0.044 | 0.754 |
|  | T3 | 33.10 | 7.75 | 34.50 | 7.20 | 32.20 | 7.63 | 0.039 | T3-T1 | 2.000E-06 | 0.076 | 8.000E-06 |
| Valine | T1 | 141.00 | 31.75 | 138.00 | 34.50 | 141.00 | 29.00 | 0.229 | T2-T1 | 0.003 | 0.426 | 0.003 |
|  | T2 | 137.00 | 28.50 | 136.00 | 25.50 | 138.00 | 34.00 | 0.689 | T3-T2 | 3.487E-08 | 1.200E-05 | 2.690E-04 |
|  | T3 | 127.00 | 28.25 | 127.00 | 29.00 | 126.00 | 24.50 | 0.665 | T3-T1 | 9.859E-13 | 1.000E-05 | 4.577E-08 |
| Cysteine | T1 | 14.10 | 8.56 | 14.50 | 6.91 | 13.90 | 8.85 | 0.950 | T2-T1 | 1.200E-05 | 0.017 | 8.400E-05 |
|  | T2 | 12.70 | 7.81 | 13.40 | 7.02 | 11.60 | 7.14 | 0.316 | T3-T2 | 0.149 | 0.236 | 0.424 |
|  | T3 | 13.35 | 7.59 | 13.70 | 6.46 | 13.15 | 9.66 | 0.864 | T3-T1 | 9.460E-07 | 0.001 | 2.710E-04 |
| Taurine | T1 | 1.56 | 0.59 | 1.68 | 0.77 | 1.50 | 0.57 | 0.092 | T2-T1 | 5.041E-10 | 2.000E-06 | 5.500E-05 |
|  | T2 | 1.32 | 0.14 | 1.32 | 0.33 | 38.35 | 0.40 | 0.550 | T3-T2 | 0.007 | 0.302 | 0.010 |
|  | T3 | 1.23 | 0.34 | 1.27 | 0.34 | 1.21 | 0.37 | 0.357 | T3-T1 | 2.359E-09 | 7.500E-05 | 1.200E-05 |
| Sum of essential AA excluding BCAA | T1 | 205.70 | 45.55 | 204.90 | 43.75 | 206.20 | 46.78 | 0.859 | T2-T1 | 4.000E-06 | 0.003 | 2.170E-04 |
|  | T2 | 213.50 | 64.50 | 210.65 | 66.78 | 220.15 | 65.75 | 0.488 | T3-T2 | 0.008 | 0.023 | 0.166 |
|  | T3 | 227.00 | 66.23 | 230.30 | 70.70 | 221.80 | 65.28 | 0.977 | T3-T1 | 4.448E-10 | 3.000E-06 | 1.000E-05 |
| Sum of essential AA including BCAA | T1 | 472.50 | 81.65 | 468.40 | 87.53 | 472.60 | 122.40 | 0.554 | T2-T1 | 0.743 | 0.952 | 0.952 |
|  | T2 | 473.90 | 101.20 | 456.85 | 97.15 | 484.50 | 109.13 | 0.632 | T3-T2 | 0.141 | 0.128 | 0.128 |
|  | T3 | 464.60 | 95.28 | 469.00 | 116.70 | 463.05 | 92.88 | 0.750 | T3-T1 | 0.292 | 0.189 | 0.189 |
| Sum of non-essential AA | T1 | 1192.86 | 209.98 | 1169.25 | 247.43 | 1199.30 | 219.04 | 0.336 | T2-T1 | 0.060 | 0.224 | 0.238 |
|  | T2 | 1168.90 | 193.37 | 1127.18 | 179.68 | 1198.54 | 210.94 | 0.016 | T3-T2 | 0.562 | 0.426 | 0.853 |
|  | T3 | 1156.40 | 221.39 | 1129.50 | 176.71 | 1190.63 | 243.28 | 0.056 | T3-T1 | 0.151 | 0.148 | 0.613 |
| Sum of BCAA | T1 | 256.50 | 59.48 | 250.05 | 65.53 | 259.60 | 55.20 | 0.202 | T2-T1 | 4.590E-04 | 0.217 | 0.001 |
|  | T2 | 249.05 | 51.33 | 245.85 | 55.38 | 250.10 | 50.90 | 0.860 | T3-T2 | 3.000E-06 | 0.002 | 4.420E-04 |
|  | T3 | 233.55 | 53.38 | 238.70 | 50.20 | 232.25 | 51.43 | 0.617 | T3-T1 | 4.901E-11 | 1.200E-04 | 1.546E-07 |
| AA, amino acids; BCAA, branched-chain amino acids; IQR, inter-quartile range. ^†^P-values represent difference in median metabolite values within a given trimester between ethnic groups, calculated by Mann-Whitney U test. ^‡^P-values represent difference in median metabolite values between paired trimesters, calculated by Wilcoxon Rank test separately for total population, Hispanic subjects and non-Hispanic subjects. Significance set at p<0.05. Bonferroni correction for multiple comparisons (N=97*3 timepoints =291) implies values are statistically significant where p <0.00017 (1.7E-4). | | | | | | | | | | | | |

**Table B:** **Total population and ethnic-specific plasma NEFA concentrations (µmol/L) within each trimester and comparison of median values between trimesters**

|  |  | **Total Population (N=160)** | | **Hispanic Subjects (N=68)** | | **Non-Hispanic Subjects (N=91)** | | **Within-trimester Comparison^†^** | **Between-trimester Comparison^‡^** | | | |
| --- | --- | --- | --- | --- | --- | --- | --- | --- | --- | --- | --- | --- |
| **NEFA** | **Trimester** | **Median** | **IQR** | **Median** | **IQR** | **Median** | **IQR** | **P-value** | **Pair** | **Total population P-value** | **Hispanic subjects P-value** | **Non-Hispanic subjects P-value** |
| C11:0 | T1 | 0.01 | 0.02 | 0.01 | 0.02 | 0.01 | 0.02 | 0.540 | T2-T1 | 0.353 | 0.158 | 1.000 |
|  | T2 | 0.00 | 0.02 | 0.00 | 0.01 | 0.01 | 0.03 | 0.014 | T3-T2 | 0.593 | 0.896 | 0.343 |
|  | T3 | 0.00 | 0.02 | 0.00 | 0.01 | 0.01 | 0.02 | 0.050 | T3-T1 | 0.082 | 0.110 | 0.223 |
| C12:0 | T1 | 1.59 | 1.03 | 1.66 | 0.80 | 1.53 | 1.15 | 0.910 | T2-T1 | 0.226 | 0.913 | 0.078 |
|  | T2 | 1.41 | 0.94 | 1.39 | 0.85 | 1.49 | 1.01 | 0.860 | T3-T2 | 0.028 | 0.405 | 0.046 |
|  | T3 | 1.58 | 1.23 | 1.43 | 1.25 | 1.66 | 1.09 | 0.126 | T3-T1 | 0.107 | 0.550 | 0.189 |
| C14:0 | T1 | 5.09 | 4.02 | 5.43 | 3.51 | 4.77 | 4.19 | 0.258 | T2-T1 | 0.088 | 0.118 | 0.250 |
|  | T2 | 4.96 | 3.30 | 4.62 | 2.69 | 5.09 | 4.03 | 0.771 | T3-T2 | 0.009 | 0.380 | 0.010 |
|  | T3 | 5.96 | 3.95 | 5.25 | 3.40 | 6.68 | 4.02 | 0.059 | T3-T1 | 0.227 | 0.452 | 0.043 |
| C14:1 | T1 | 0.88 | 0.77 | 0.96 | 0.75 | 0.78 | 0.79 | 0.210 | T2-T1 | 0.001 | 0.002 | 0.058 |
|  | T2 | 0.80 | 0.61 | 0.81 | 0.50 | 0.71 | 0.67 | 0.890 | T3-T2 | 0.539 | 0.618 | 0.252 |
|  | T3 | 0.87 | 0.71 | 0.76 | 0.53 | 0.98 | 0.79 | 0.035 | T3-T1 | 0.039 | 4.190E-04 | 0.916 |
| C15:0 | T1 | 0.93 | 0.56 | 0.99 | 0.46 | 0.86 | 0.61 | 0.340 | T2-T1 | 0.017 | 0.044 | 0.094 |
|  | T2 | 0.86 | 0.45 | 0.84 | 0.39 | 0.89 | 0.59 | 0.905 | T3-T2 | 0.036 | 0.546 | 0.038 |
|  | T3 | 0.93 | 0.59 | 0.88 | 0.50 | 1.03 | 0.66 | 0.101 | T3-T1 | 0.870 | 0.153 | 0.411 |
| C16:0 | T1 | 64.20 | 36.80 | 68.20 | 32.65 | 55.80 | 38.05 | 0.061 | T2-T1 | 0.019 | 0.039 | 0.154 |
|  | T2 | 58.85 | 26.93 | 60.00 | 25.93 | 53.90 | 29.50 | 0.186 | T3-T2 | 0.001 | 0.113 | 0.003 |
|  | T3 | 68.35 | 32.60 | 68.90 | 31.60 | 67.30 | 33.28 | 0.587 | T3-T1 | 0.331 | 0.344 | 0.054 |
| C16:1 | T1 | 8.20 | 7.34 | 9.61 | 7.53 | 7.65 | 6.78 | 0.042 | T2-T1 | 0.003 | 0.001 | 0.228 |
|  | T2 | 7.28 | 6.10 | 7.91 | 5.35 | 7.19 | 6.60 | 0.298 | T3-T2 | 0.034 | 0.292 | 0.053 |
|  | T3 | 8.69 | 6.83 | 8.32 | 7.13 | 9.47 | 6.89 | 0.391 | T3-T1 | 0.323 | 0.006 | 0.289 |
| C17:0 | T1 | 1.35 | 0.69 | 1.41 | 0.54 | 1.28 | 0.84 | 0.133 | T2-T1 | 0.019 | 0.065 | 0.091 |
|  | T2 | 1.27 | 0.57 | 7.91 | 0.50 | 1.24 | 0.60 | 0.340 | T3-T2 | 0.021 | 0.476 | 0.023 |
|  | T3 | 1.38 | 0.66 | 1.38 | 0.59 | 1.39 | 0.75 | 0.548 | T3-T1 | 0.945 | 0.153 | 0.324 |
| C17:1 | T1 | 0.67 | 0.50 | 0.74 | 0.40 | 0.58 | 0.55 | 0.035 | T2-T1 | 0.003 | 0.003 | 0.120 |
|  | T2 | 0.60 | 0.40 | 0.63 | 0.37 | 0.60 | 0.40 | 0.446 | T3-T2 | 0.064 | 0.642 | 0.060 |
|  | T3 | 0.67 | 0.47 | 0.65 | 0.45 | 0.68 | 0.50 | 0.365 | T3-T1 | 0.156 | 0.001 | 0.548 |
| C18:0 | T1 | 25.00 | 11.30 | 25.00 | 8.90 | 25.60 | 13.80 | 0.265 | T2-T1 | 0.243 | 0.368 | 0.346 |
|  | T2 | 24.35 | 11.25 | 25.35 | 9.10 | 23.40 | 12.30 | 0.194 | T3-T2 | 0.004 | 0.450 | 0.004 |
|  | T3 | 26.35 | 11.48 | 25.70 | 11.60 | 27.45 | 11.68 | 0.507 | T3-T1 | 0.061 | 0.770 | 0.043 |
| C18:1 | T1 | 93.80 | 59.60 | 101.00 | 48.20 | 86.70 | 65.40 | 0.101 | T2-T1 | 0.008 | 0.012 | 0.139 |
|  | T2 | 83.95 | 52.90 | 84.65 | 48.15 | 79.00 | 48.90 | 0.376 | T3-T2 | 0.011 | 0.203 | 0.022 |
|  | T3 | 97.50 | 53.80 | 93.40 | 57.50 | 100.50 | 52.68 | 0.360 | T3-T1 | 0.827 | 0.068 | 0.292 |
| C18:2 | T1 | 44.20 | 28.20 | 48.10 | 28.50 | 39.80 | 28.95 | 0.019 | T2-T1 | 0.002 | 0.012 | 0.032 |
|  | T2 | 38.75 | 23.25 | 41.00 | 18.05 | 35.20 | 24.60 | 0.056 | T3-T2 | 0.259 | 0.734 | 0.262 |
|  | T3 | 41.95 | 22.50 | 43.40 | 21.40 | 39.95 | 23.85 | 0.706 | T3-T1 | 0.112 | 0.017 | 0.801 |
| C18:3 | T1 | 4.19 | 2.85 | 4.52 | 2.89 | 3.79 | 2.41 | 0.095 | T2-T1 | 0.006 | 0.018 | 0.076 |
|  | T2 | 3.75 | 2.24 | 3.77 | 1.92 | 3.69 | 2.87 | 0.422 | T3-T2 | 0.332 | 0.679 | 0.351 |
|  | T3 | 4.13 | 2.41 | 4.06 | 2.20 | 4.30 | 2.67 | 0.518 | T3-T1 | 0.169 | 0.020 | 0.918 |
| C20:1 | T1 | 0.76 | 0.55 | 0.80 | 0.47 | 0.73 | 0.49 | 0.069 | T2-T1 | 0.090 | 0.021 | 0.634 |
|  | T2 | 0.74 | 0.45 | 0.79 | 0.42 | 0.72 | 0.50 | 0.410 | T3-T2 | 0.003 | 0.069 | 0.026 |
|  | T3 | 0.86 | 0.46 | 0.82 | 0.36 | 0.88 | 0.50 | 0.624 | T3-T1 | 0.228 | 0.527 | 0.063 |
| C20:2 | T1 | 0.74 | 0.45 | 0.86 | 0.46 | 0.66 | 0.42 | 0.003 | T2-T1 | 0.005 | 0.009 | 0.095 |
|  | T2 | 0.68 | 0.45 | 0.71 | 0.44 | 0.60 | 0.46 | 0.010 | T3-T2 | 0.765 | 0.528 | 0.387 |
|  | T3 | 0.71 | 0.41 | 0.58 | 0.41 | 0.71 | 0.45 | 0.471 | T3-T1 | 0.045 | 0.009 | 0.678 |
| C20:3 | T1 | 0.68 | 0.41 | 0.81 | 0.41 | 0.63 | 0.41 | 0.019 | T2-T1 | 9.000E-06 | 4.910E-04 | 0.001 |
|  | T2 | 0.59 | 0.34 | 0.62 | 0.28 | 0.56 | 0.35 | 0.096 | T3-T2 | 0.777 | 0.392 | 0.774 |
|  | T3 | 0.59 | 0.32 | 0.58 | 0.27 | 0.60 | 0.34 | 0.953 | T3-T1 | 5.300E-05 | 3.140E-04 | 0.027 |
| C20:4 | T1 | 1.63 | 0.98 | 1.64 | 0.93 | 1.62 | 0.97 | 0.708 | T2-T1 | 1.186E-11 | 2.000E-06 | 4.341E-07 |
|  | T2 | 1.25 | 0.77 | 1.19 | 0.48 | 1.25 | 0.82 | 0.559 | T3-T2 | 0.214 | 0.268 | 0.484 |
|  | T3 | 1.21 | 0.75 | 1.14 | 0.55 | 1.35 | 0.88 | 0.067 | T3-T1 | 1.225E-11 | 1.000E-06 | 1.000E-06 |
| C20:5 | T1 | 0.11 | 0.10 | 0.10 | 0.08 | 0.14 | 0.13 | 0.031 | T2-T1 | 2.151E-08 | 1.100E-05 | 8.300E-05 |
|  | T2 | 0.10 | 0.08 | 0.08 | 0.06 | 0.11 | 0.09 | 0.003 | T3-T2 | 0.502 | 0.768 | 0.509 |
|  | T3 | 0.09 | 0.10 | 0.07 | 0.06 | 0.12 | 0.11 | 0.004 | T3-T1 | 2.000E-06 | 0.001 | 1.250E-04 |
| C22:4 | T1 | 0.39 | 0.23 | 0.42 | 0.23 | 0.34 | 0.23 | 0.032 | T2-T1 | 7.000E-06 | 2.430E-04 | 0.003 |
|  | T2 | 0.32 | 0.19 | 0.33 | 0.19 | 0.31 | 0.20 | 0.341 | T3-T2 | 0.753 | 0.310 | 0.642 |
|  | T3 | 0.32 | 0.19 | 0.31 | 0.13 | 0.34 | 0.23 | 0.370 | T3-T1 | 6.800E-05 | 7.700E-05 | 0.060 |
| C22:5 | T1 | 0.55 | 0.34 | 0.56 | 0.31 | 0.53 | 0.38 | 0.567 | T2-T1 | 2.000E-06 | 1.710E-04 | 0.001 |
|  | T2 | 0.45 | 0.27 | 0.45 | 0.22 | 0.43 | 0.35 | 0.643 | T3-T2 | 0.718 | 0.610 | 0.936 |
|  | T3 | 0.45 | 0.28 | 0.41 | 0.22 | 0.48 | 0.35 | 0.127 | T3-T1 | 5.200E-05 | 0.001 | 0.014 |
| C22:6 | T1 | 1.25 | 0.93 | 1.16 | 0.63 | 1.30 | 1.16 | 0.144 | T2-T1 | 0.001 | 0.002 | 0.042 |
|  | T2 | 1.15 | 0.62 | 1.01 | 0.47 | 1.30 | 0.86 | 0.005 | T3-T2 | 0.832 | 0.777 | 0.906 |
|  | T3 | 1.08 | 0.81 | 0.95 | 0.66 | 1.26 | 1.04 | 0.004 | T3-T1 | 0.002 | 0.007 | 0.055 |
| Sum of saturated NEFA | T1 | 99.43 | 51.00 | 103.31 | 46.31 | 89.16 | 58.49 | 0.105 | T2-T1 | 0.043 | 0.095 | 0.162 |
|  | T2 | 91.90 | 43.71 | 96.43 | 37.09 | 87.29 | 50.55 | 0.218 | T3-T2 | 0.002 | 0.170 | 0.004 |
|  | T3 | 106.16 | 50.94 | 106.01 | 47.08 | 106.14 | 51.54 | 0.440 | T3-T1 | 0.191 | 0.561 | 0.043 |
| Sum of monounsaturated NEFA | T1 | 103.82 | 66.25 | 112.55 | 54.28 | 94.35 | 70.52 | 0.068 | T2-T1 | 0.004 | 0.008 | 0.094 |
|  | T2 | 91.82 | 57.69 | 94.42 | 55.28 | 90.36 | 55.28 | 0.291 | T3-T2 | 0.011 | 0.287 | 0.015 |
|  | T3 | 108.17 | 62.57 | 102.96 | 60.62 | 114.25 | 61.98 | 0.285 | T3-T1 | 0.694 | 0.024 | 0.249 |
| Sum of polyunsaturated NEFA | T1 | 53.49 | 31.99 | 57.48 | 31.97 | 50.27 | 31.56 | 0.023 | T2-T1 | 0.001 | 0.008 | 0.011 |
|  | T2 | 47.00 | 28.15 | 49.39 | 21.20 | 43.17 | 29.29 | 0.082 | T3-T2 | 0.218 | 0.728 | 0.197 |
|  | T3 | 50.15 | 26.31 | 51.70 | 24.44 | 49.56 | 27.68 | 0.937 | T3-T1 | 0.075 | 0.010 | 0.804 |
| IQR, inter-quartile range; NEFA, non-esterified fatty acids. ^†^P-values represent difference in median metabolite values within a given trimester between ethnic groups, calculated by Mann-Whitney U test. ^‡^P-values represent difference in median metabolite values between paired trimesters, calculated by Wilcoxon Rank test separately for total population, Hispanic subjects and non-Hispanic subjects. Significance set at p<0.05. Bonferroni correction for multiple comparisons (N=97*3 timepoints =291) implies values are statistically significant where p <0.00017 (1.7E-4). | | | | | | | | | | | | |

**Table C: Total population and ethnic-specific metabolic ratios as indicators of BCAA and fatty acid metabolism within each trimester and comparison of median values between trimesters**

|  |  |  | **Total Population (N=160)** | | **Hispanic Subjects (N=68)** | | **Non-Hispanic Subjects (N=91)** | | **Within-trimester Comparison^†^** | **Between-trimester Comparison^‡^** | | | |
| --- | --- | --- | --- | --- | --- | --- | --- | --- | --- | --- | --- | --- | --- |
| **Ratio** | **Interpretation** | **Trimester** | **Median** | **IQR** | **Median** | **IQR** | **Median** | **IQR** | **p-value** | **Pair** | **Total population P-value** | **Hispanic subjects P-value** | **Non-Hispanic subjects P-value** |
| Carn.a.C14/Carn | CPT-1 rate | T1 | 0.001 | 0.0008 | 0.001 | 0.0007 | 0.001 | 0.0008 | 0.943 | T2-T1 | 0.378 | 0.615 | 0.200 |
|  |  | T2 | 0.002 | 0.0002 | 0.002 | 0.0003 | 0.001 | 0.0008 | 0.546 | T3-T2 | 4.457E-07 | 1.260E-04 | 0.001 |
|  |  | T3 | 0.002 | 0.0010 | 0.002 | 0.0001 | 0.002 | 0.0010 | 0.407 | T3-T1 | 5.077E-07 | 0.001 | 3.700E-04 |
| Carn.a.C16/Carn | CPT-1 rate | T1 | 0.003 | 0.0001 | 0.003 | 0.0009 | 0.003 | 0.0012 | 0.597 | T2-T1 | 7.974E-08 | 1.700E-05 | 0.001 |
|  |  | T2 | 0.003 | 0.0012 | 0.003 | 0.0013 | 0.003 | 0.0012 | 0.221 | T3-T2 | 2.000E-06 | 0.013 | 4.200E-05 |
|  |  | T3 | 0.004 | 0.0018 | 0.004 | 0.0017 | 0.004 | 0.0019 | 0.402 | T3-T1 | 7.803E-14 | 1.500E-05 | 2.443E-09 |
| Carn.a.C18/Carn | CPT-1 rate | T1 | 0.001 | 0.0007 | 0.001 | 0.0007 | 0.001 | 0.0007 | 0.388 | T2-T1 | 5.818E-07 | 8.800E-05 | 0.002 |
|  |  | T2 | 0.001 | 0.0008 | 0.001 | 0.0008 | 0.005 | 0.0007 | 0.792 | T3-T2 | 0.058 | 0.277 | 0.167 |
|  |  | T3 | 0.002 | 0.0009 | 0.001 | 0.0007 | 0.006 | 0.0009 | 0.344 | T3-T1 | 1.210E-08 | 2.720E-04 | 2.100E-05 |
| Carn.a.C18.1/Carn | CPT-1 rate | T1 | 0.004 | 0.0022 | 0.004 | 0.0019 | 0.004 | 0.0023 | 0.959 | T2-T1 | 7.000E-06 | 0.011 | 3.710E-04 |
|  |  | T2 | 0.005 | 0.0021 | 0.005 | 0.0020 | 0.005 | 0.0020 | 0.321 | T3-T2 | 1.900E-05 | 0.006 | 0.001 |
|  |  | T3 | 0.006 | 0.0025 | 0.005 | 0.0020 | 0.006 | 0.0029 | 0.010 | T3-T1 | 1.967E-11 | 3.04E-04 | 3.574E-08 |
| Carn.a.C18.2/Carn | CPT-1 rate | T1 | 0.003 | 0.0017 | 0.003 | 0.0016 | 0.003 | 0.0018 | 0.654 | T2-T1 | 0.001 | 0.007 | 0.030 |
|  |  | T2 | 0.004 | 0.0019 | 0.004 | 0.019 | 0.004 | 0.0018 | 0.357 | T3-T2 | 0.005 | 0.361 | 0.004 |
|  |  | T3 | 0.004 | 0.0022 | 0.004 | 0.0019 | 0.004 | 0.0022 | 0.285 | T3-T1 | 2.245E-08 | 0.005 | 3.000E-06 |
| Carn.a.C2/Carn.a.C14 | β-Oxidation | T1 | 145.40 | 68.75 | 153.54 | 73.27 | 134.95 | 68.42 | 0.323 | T2-T1 | 0.429 | 0.546 | 0.136 |
|  |  | T2 | 137.43 | 63.14 | 145.15 | 72.48 | 134.30 | 65.58 | 0.126 | T3-T2 | 0.001 | 0.018 | 0.019 |
|  |  | T3 | 115.29 | 54.51 | 122.97 | 48.52 | 114.40 | 56.92 | 0.407 | T3-T1 | 3.200E-05 | 0.020 | 0.001 |
| Carn.a.C2/Carn.a.C16 | β-Oxidation | T1 | 74.80 | 28.92 | 74.24 | 28.28 | 74.84 | 28.33 | 0.744 | T2-T1 | 1.222E-07 | 6.700E-05 | 3.410E-04 |
|  |  | T2 | 61.79 | 25.27 | 64.38 | 23.05 | 60.86 | 28.23 | 0.656 | T3-T2 | 0.005 | 0.119 | 0.017 |
|  |  | T3 | 56.53 | 20.73 | 58.31 | 22.92 | 55.38 | 18.49 | 0.407 | T3-T1 | 2.703E-12 | 2.600E-05 | 4.059E-08 |
| Carn.a.C2/Carn.a.C18 | β-Oxidation | T1 | 186.50 | 110.20 | 195.52 | 117.19 | 181.21 | 95.52 | 0.094 | T2-T1 | 8.100E-05 | 0.002 | 0.015 |
|  |  | T2 | 161.88 | 85.21 | 166.67 | 85.42 | 156.50 | 78.93 | 0.402 | T3-T2 | 0.658 | 0.708 | 0.912 |
|  |  | T3 | 148.76 | 79.30 | 164.84 | 76.36 | 144.73 | 77.10 | 0.237 | T3-T1 | 2.200E-05 | 0.001 | 0.009 |
| Carn.a.C2/Carn.a.C18.1 | β-Oxidation | T1 | 48.44 | 18.82 | 50.73 | 18.32 | 47.43 | 21.78 | 0.339 | T2-T1 | 1.320E-07 | 0.010 | 4.000E-06 |
|  |  | T2 | 40.70 | 15.50 | 44.85 | 15.86 | 39.40 | 13.77 | 0.015 | T3-T2 | 0.014 | 0.084 | 0.101 |
|  |  | T3 | 38.81 | 18.50 | 41.26 | 15.00 | 35.91 | 20.10 | 0.014 | T3-T1 | 1.095E-09 | 0.001 | 2.292E-07 |
| Carn.a.C2/Carn.a.C18.2 | β-Oxidation | T1 | 62.19 | 28.84 | 64.61 | 21.73 | 58.36 | 33.00 | 0.262 | T2-T1 | 3.452E-04 | 0.013 | 0.021 |
|  |  | T2 | 53.92 | 30.26 | 56.89 | 26.14 | 52.92 | 31.30 | 0.933 | T3-T2 | 0.089 | 0.680 | 0.065 |
|  |  | T3 | 51.72 | 28.56 | 53.90 | 28.03 | 50.14 | 29.43 | 0.307 | T3-T1 | 2.300E-05 | 0.028 | 2.650E-04 |
| Carn, acylcarnitine; CPT, carnitine palmitoyl transferase; IQR, inter-quartile range. ^†^P-values represent difference in median metabolite values within a given trimester between ethnic groups, calculated by Mann-Whitney U test. ^‡^P-values represent difference in median metabolite values between paired trimesters, calculated by Wilcoxon Rank test separately for total population, Hispanic subjects and non-Hispanic subjects. Significance set at p<0.05. Bonferroni correction for multiple comparisons (N=97*3 timepoints =291) implies values are statistically significant where p <0.00017 (1.7E-4). | | | | | | | | | | | | | |

**Table D: Total population and ethnic-specific plasma concentrations (µmol/L) of metabolites generated through processes of ketogenesis and oxidation of glucose, amino acids and fatty acids, within each trimester and comparison of median values between trimesters**

|  |  | **Total Population (N=160)** | | **Hispanic Subjects (N=68)** | | **Non-Hispanic Subjects (N=91)** | | **Within-trimester Comparison^†^** | **Between-trimester Comparison^‡^** | | | |
| --- | --- | --- | --- | --- | --- | --- | --- | --- | --- | --- | --- | --- |
| **Metabolite** | **Trimester** | **Median** | **IQR** | **Median** | **IQR** | **Median** | **IQR** | **P-value** | **Pair** | **Total population P-value** | **Hispanic subjects P-value** | **Non-Hispanic subjects P-value** |
| Citric acid | T1 | 5.23 | 1.01 | 5.84 | 1.92 | 5.33 | 2.02 | 0.007 | T2-T1 | 2.700E-05 | 0.015 | 0.001 |
|  | T2 | 6.29 | 2.58 | 6.72 | 2.60 | 6.16 | 2.42 | 0.136 | T3-T2 | 4.850E-09 | 1.200E-05 | 8.400E-05 |
|  | T3 | 7.49 | 2.83 | 7.84 | 2.64 | 7.43 | 2.56 | 0.093 | T3-T1 | 8.395E-16 | 6.642E-07 | 4.711E-10 |
| Isocitric acid | T1 | 0.07 | 0.09 | 0.06 | 0.09 | 0.07 | 0.09 | 0.820 | T2-T1 | 0.332 | 0.192 | 0.821 |
|  | T2 | 0.08 | 0.08 | 0.10 | 0.09 | 0.07 | 0.08 | 0.104 | T3-T2 | 0.001 | 0.024 | 0.013 |
|  | T3 | 0.10 | 0.10 | 0.11 | 0.10 | 0.10 | 0.09 | 0.373 | T3-T1 | 1.180E-04 | 0.001 | 0.024 |
| Alpha-ketoglutaric acid | T1 | 1.87 | 0.56 | 1.94 | 0.61 | 1.80 | 0.53 | 0.477 | T2-T1 | 0.022 | 0.090 | 0.171 |
|  | T2 | 2.01 | 0.65 | 2.07 | 0.59 | 1.93 | 0.68 | 0.178 | T3-T2 | 0.115 | 0.870 | 0.042 |
|  | T3 | 2.03 | 0.53 | 2.07 | 0.92 | 2.02 | 0.55 | 0.896 | T3-T1 | 1.680E-04 | 0.050 | 0.002 |
| Succinic acid | T1 | 3.87 | 1.30 | 3.81 | 1.13 | 3.96 | 1.34 | 0.594 | T2-T1 | 0.002 | 0.003 | 0.122 |
|  | T2 | 3.63 | 1.24 | 3.41 | 1.58 | 3.74 | 1.14 | 0.049 | T3-T2 | 0.872 | 0.914 | 0.830 |
|  | T3 | 3.60 | 1.25 | 3.32 | 1.42 | 3.62 | 1.25 | 0.045 | T3-T1 | 0.035 | 0.014 | 0.557 |
| Fumaric acid | T1 | 0.17 | 0.06 | 0.17 | 0.05 | 0.17 | 0.08 | 0.888 | T2-T1 | 0.002 | 0.069 | 0.012 |
|  | T2 | 0.18 | 0.07 | 0.18 | 0.08 | 0.18 | 0.07 | 0.602 | T3-T2 | 2.530E-04 | 0.003 | 0.033 |
|  | T3 | 0.21 | 0.09 | 0.21 | 0.09 | 0.21 | 0.09 | 0.736 | T3-T1 | 4.438E-07 | 0.002 | 1.270E-04 |
| Malic acid | T1 | 0.48 | 0.16 | 0.51 | 0.17 | 0.47 | 0.15 | 0.722 | T2-T1 | 0.150 | 0.676 | 0.147 |
|  | T2 | 0.49 | 0.14 | 0.49 | 0.17 | 0.51 | 0.13 | 0.306 | T3-T2 | 1.593E-08 | 2.200E-05 | 1.870E-05 |
|  | T3 | 0.56 | 0.21 | 0.57 | 0.21 | 0.56 | 0.21 | 0.725 | T3-T1 | 8.787E-09 | 3.820E-04 | 7.000E-06 |
| Pyruvic acid | T1 | 303.00 | 123.00 | 312.50 | 121.75 | 295.00 | 116.00 | 0.193 | T2-T1 | 0.511 | 0.496 | 0.186 |
|  | T2 | 293.50 | 118.50 | 292.00 | 116.50 | 294.00 | 120.00 | 0.897 | T3-T2 | 0.032 | 0.116 | 0.174 |
|  | T3 | 318.50 | 113.25 | 323.50 | 104.50 | 315.00 | 120.00 | 0.632 | T3-T1 | 0.001 | 0.154 | 0.004 |
| Lactic acid | T1 | 1690.00 | 605.00 | 1730.00 | 590.00 | 1680.00 | 635.00 | 0.766 | T2-T1 | 0.297 | 0.459 | 0.041 |
|  | T2 | 1675.00 | 645.00 | 1365.00 | 690.00 | 1700.00 | 630.00 | 0.320 | T3-T2 | 0.048 | 0.210 | 0.144 |
|  | T3 | 1820.00 | 652.50 | 1750.00 | 585.00 | 1890.00 | 700.00 | 0.522 | T3-T1 | 0.001 | 0.098 | 0.003 |
| Methyl malonic acid | T1 | 0.41 | 0.22 | 0.40 | 0.22 | 0.42 | 0.22 | 0.493 | T2-T1 | 0.308 | 0.178 | 0.828 |
|  | T2 | 0.39 | 0.11 | 0.37 | 0.28 | 0.39 | 0.21 | 0.440 | T3-T2 | 0.166 | 0.183 | 0.463 |
|  | T3 | 0.41 | 0.23 | 0.40 | 0.24 | 0.41 | 0.22 | 0.749 | T3-T1 | 0.750 | 0.664 | 0.589 |
| 3-methyl-2-oxobutanoic acid | T1 | 32.95 | 8.25 | 33.50 | 9.55 | 31.40 | 7.60 | 0.091 | T2-T1 | 0.003 | 0.011 | 0.045 |
|  | T2 | 30.00 | 8.30 | 30.45 | 8.28 | 29.75 | 8.43 | 0.311 | T3-T2 | 0.332 | 0.694 | 0.283 |
|  | T3 | 30.10 | 9.18 | 31.30 | 8.20 | 28.75 | 8.50 | 0.040 | T3-T1 | 0.006 | 0.099 | 0.012 |
| 3-methyl-2-oxovalveric acid | T1 | 41.60 | 10.55 | 38.80 | 13.60 | 41.90 | 8.88 | 0.929 | T2-T1 | 4.000E-06 | 0.001 | 0.002 |
|  | T2 | 38.50 | 11.70 | 38.50 | 9.35 | 38.35 | 11.88 | 0.548 | T3-T2 | 0.893 | 0.926 | 0.636 |
|  | T3 | 39.30 | 11.20 | 39.40 | 11.60 | 38.90 | 10.60 | 0.608 | T3-T1 | 0.001 | 0.079 | 0.001 |
| 4-methyl-2-oxovalveric acid | T1 | 67.10 | 15.30 | 64.50 | 17.00 | 69.00 | 14.80 | 0.445 | T2-T1 | 7.846E-09 | 2.330E-04 | 3.000E-06 |
|  | T2 | 59.30 | 14.70 | 57.35 | 12.93 | 60.30 | 15.45 | 0.372 | T3-T2 | 0.724 | 0.753 | 0.503 |
|  | T3 | 59.35 | 14.53 | 59.90 | 13.00 | 57.45 | 16.48 | 0.480 | T3-T1 | 4.499E-07 | 0.005 | 7.000E-06 |
| Alpha-aminoadipic acid | T1 | 0.17 | 0.03 | 0.18 | 0.07 | 0.17 | 0.07 | 0.121 | T2-T1 | 0.909 | 0.478 | 0.303 |
|  | T2 | 0.18 | 0.06 | 0.18 | 0.06 | 0.17 | 0.06 | 0.480 | T3-T2 | 0.283 | 0.717 | 0.313 |
|  | T3 | 0.18 | 0.07 | 0.19 | 0.06 | 0.18 | 0.07 | 0.747 | T3-T1 | 0.210 | 0.589 | 0.279 |
| Beta-hydroxybutyric acid | T1 | 47.85 | 53.50 | 47.50 | 56.10 | 48.85 | 50.48 | 0.224 | T2-T1 | 0.869 | 0.739 | 0.950 |
|  | T2 | 50.00 | 39.60 | 53.40 | 41.40 | 42.95 | 36.95 | 0.138 | T3-T2 | 3.000E-06 | 0.021 | 5.100E-05 |
|  | T3 | 66.80 | 68.33 | 65.65 | 48.73 | 66.20 | 72.15 | 0.838 | T3-T1 | 0.001 | 0.058 | 0.006 |
| IQR, inter-quartile range. ^†^P-values represent difference in median metabolite values within a given trimester between ethnic groups, calculated by Mann-Whitney U test. ^‡^P-values represent difference in median metabolite values between paired trimesters, calculated by Wilcoxon Rank test separately for total population, Hispanic subjects and non-Hispanic subjects. Significance set at p<0.05. Bonferroni correction for multiple comparisons (N=97*3 timepoints =291) implies values are statistically significant where p <0.00017 (1.7E-4). | | | | | | | | | | | | |
